# Geometric Aberration Theory of Offner Imaging Spectrometers

**DOI:** 10.3390/s19184046

**Published:** 2019-09-19

**Authors:** Meihong Zhao, Yanxiu Jiang, Shuo Yang, Wenhao Li

**Affiliations:** 1 Changchun Institute of Optics, Fine Mechanics and Physics, Chinese Academy of Sciences, Changchun 130033, China; zmh_optics@126.com (M.Z.);; 2 University of Chinese Academy of Sciences, Beijing 100049, China

**Keywords:** aberration theory, Offner imaging spectrometer, convex grating, spot diagrams

## Abstract

A third-order aberration theory has been developed for the Offner imaging spectrometer comprising an extended source; two concave mirrors; a convex diffraction grating; and an image plane. Analytic formulas of the spot diagram are derived for tracing rays through the system based on Fermat’s principle. The proposed theory can be used to discuss in detail individual aberrations of the system such as coma, spherical aberration and astigmatism, and distortion together with the focal conditions. It has been critically evaluated as well in a comparison with exact ray tracing constructed using the commercial software ZEMAX. In regard to the analytic formulas, the results show a high degree of practicality.

## 1. Introduction

An imaging spectrometer can provide a simultaneous collection of spatial and spectral information of targets with high resolution [1]. Currently, spectrometers have become an indispensable part of many fields including satellite remote sensing, space exploration, security, environment assessment, resource detection, agriculture, medicine, manufacturing, oceanography, and ecology [2,3,4,5,6].

The recent trend in imaging spectrometers is toward a simple set-up and a very compact configuration with high optical performance over the whole spectral range of the system [7]. This can be observed in the Offner imaging spectrometer with a concentric structure, using spherical optics. This spectrometer is obtained by replacing the convex secondary mirror of the Offner imaging system with a reflective convex diffraction grating [8]. It provides a high signal-to-noise ratio and small spot sizes together with low spatial and spectral distortions [7,8,9,10,11]. Because diffraction occurs at the grating, the perfect symmetry of the concentric configuration is altered, thereby increasing for example the coma and astigmatism. Although good optical performance is maintained with the rapid development of imaging spectrometers; more improvements need to be achieved to meet the dual demands for higher spatial and spectral resolution.

There have been various attempts to optimize and design an aberration-correct Offner imaging spectrometer. In 1999, Chrisp split the concave mirror into two concentric mirrors of different radii, increasing the degrees-of-freedom of the system designs [12]. By changing the off-axis parameters, tilting or decentering some elements, and making appropriate adjustments to the radii of the two spherical mirrors, the optical quality of the system was optimized. In 2001, Xiang and Mikes proposed an aberration-corrected spectrometer that included a convex diffraction grating having a number of nonparallel lines [13]. They believed the curves of the convex grating provided the correction for field aberrations. However, forming such a convex grating is difficult with the existing technology and theory. In 2006, Prieto-Blanco and coworkers presented an approach based on the calculation of both the meridional and the sagittal images of an off-axis object point [5]. Making the meridional and sagittal curves tangent to each other for a given wavelength results in a decrease in astigmatism. In 2007, Robert analyzed the out-of-plane dispersion in an Offner spectrometer. When the dispersion is perpendicular to the meridional plane, better performance is obtained for the system with a short entrance slit [14]. In 2014, Prieto-Blanco and coworkers proposed a Wynne-Offner layout consisting of a concave mirror and a concentric meniscus lens that included a diffraction grating at the center of one of its surfaces [15,16,17,18]. All the above methods have described the effect of aberrations such as astigmatism on the optical quality of the Offner spectrometer and how to optimize the system. However, these methods are relatively singular-use solutions and are not widely used in developing a system for different requirements [19,20].

In this paper, we propose a third-order geometric aberration theory of the Offner imaging spectrometer to provide an alternative aberration-correction method. This method is an extension and new application of Namioka’s theories [21,22,23,24,25,26]. Namioka and his team have shown aberration theories based on the light path function for a single grating or a double-element system that can correctly describe the individual aberrations and can be used to design an advanced optical system. Taking an extended source into consideration, analytic formulas of the spot diagram and the individual aberrations are derived for tracing rays through the system based on Fermat’s principle and Namioka’s theories. With these formulas, aberrations including coma, aberration, astigmatism, and distortion of the three-concentric-element (Offner) configuration are discussed in detail together with focal conditions. Finally, the theory is critically evaluated in a comparison with exact ray tracing constructed using the commercial software ZEMAX (Zemax software development company, bellevue, WS, USA). The results indicate a high degree of validity of the analytic formulas.

## 2. Three-Concentric-Element (Offner) Optical System

**Figure 1:**
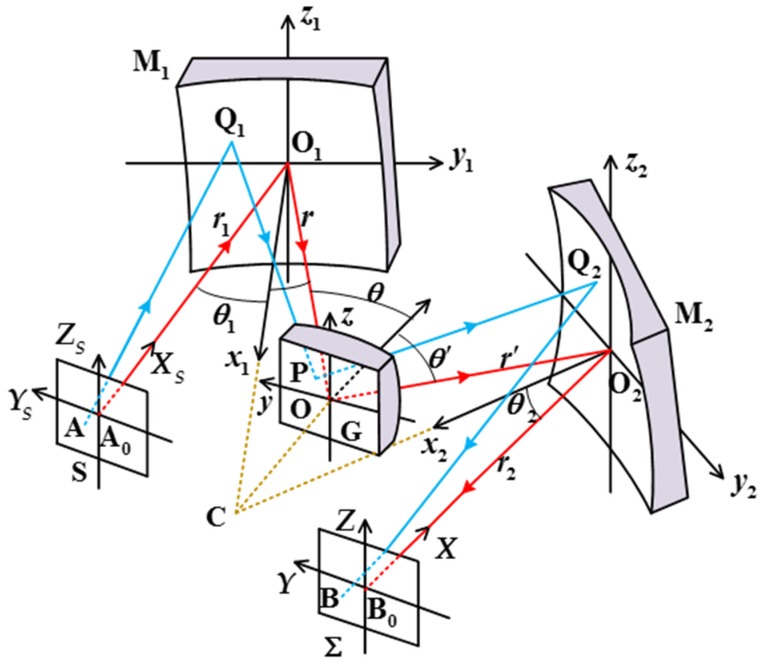
Schematic diagram of an Offner configuration and its coordinate systems.

We consider an Offner optical system that comprises a planar light source S, two concave mirrors M_1_ and M_2_, a convex diffraction grating G, and an image plane Σ (Figure 1). In this system, the elements are arranged in such a way that the normal axes to S at A_0_, to M_1_ at O_1_, to G at O, and to M_2_ at O_2_ lie in a common plane called the meridional plane. The incident principal ray A_0_O_1_ is reflected by M_1_ toward O, and the reflected principal ray O_1_O of wavelength λ in the m_1_th order is diffracted by G toward O_2_. The diffracted principal ray OO_2_ is then further reflected by M_2_. This reflected principal ray of λ meets Σ at a point B_0_, which lies in the meridional plane as well. Here we assume that the principal ray of wavelength λ is designed to end up in the center of the image plane Σ; and we assume that the image plane Σ is perpendicular both to the reflected principal ray O_2_B_0_ and to the meridional plane as well. The distances A_0_O_1_, O_1_O, OO_2_, and O_2_B_0_ are denoted by *r*_1_, *r*, *r*′, and *r*_2_, respectively.

For convenience, we introduce five rectangular coordinate systems attached to S, M_1_, G, M_2_, and Σ (Figure 1). The origins are at A_0_, O_1_, O, O_2_, and B_0_, the X_S_, x_1_, x, x_2_, and X axes are the normal axes of the respective elements, and the Y_S_, y_1_, y, y_2_, and Y axes lie in the meridional plane. A general ray originating from a source point A in S is reflected at a point Q_1_ on the surface of M_1_. The reflected ray meets G at a point P on the *n*th groove of G, and the diffracted ray of wavelength λ in *m*-th order meets M_2_ at a point Q_2_. The outgoing ray of wavelength λ from Q_2_ intersects Σ at a point B, forming a spot in the image plane Σ. We designate the coordinates of A, Q_1_, P, Q_2_, and B by (0, s, z), (*ξ*_1_, *ω*_1_, *l*_1_), (*ξ*, *ω*, *l*), (*ξ*_2_, *ω*_2_, *l*_2_), and (0, Y, Z) in the X_S_Y_S_Z_S_, *x*_1_*y*_1_*z*_1_, xyz, x_2_y_2_z_2_, and XYZ systems, respectively, and those of A and B as well by (*x*_1_, *y*_1_, *z*_1_) and (*x*_2_, *y*_2_, *z*_2_) in the *x*_1_*y*_1_*z*_1_ and x_2_y_2_z_2_ system, separately. Here, *x*_1_ and *y*_1_ are expressed as

(1) $$x_{1}=r_{1}\cos\theta_{1}+s\sin\theta_{1},\;y_{1}=r_{1}\sin\theta_{1}-s\cos\theta_{1}.$$

We assume as well that the zeroth groove of G passes through O, and that the groove number *n* is positive or negative according to whether the *n*-th groove passes through the y-axis on its positive or negative side. The groove number *n* of G expressed in a power series of *ω* and *l* is given as [26]:

(2) $$n\lambda_{0}=n_{10}\omega +\frac{1}{2}(n_{20}\omega^{2}+n_{02}l^{2}+n_{30}\omega^{3}+n_{12}\omega l^{2})+\frac{1}{8}(n_{40}\omega^{4}+2n_{22}\omega^{2}l^{2}+n_{04}l^{4})+\ldots ,$$

where λ_0_ is the recording wavelength of G.

In this system shown in Figure 1, both the concave mirrors M_1_ and M_2_ and the convex grating G are spherical in shape. The corresponding mathematical expression of the surface figure of M_i_ (or G) is given by

(3) $${\left(\xi_{i}-R_{i}\right)}^{2}+\omega_{i}{}^{2}+l_{i}{}^{2}=R_{i}{}^{2},$$

where *R_i_* (*i* = 1, 2 for M_1_ and M_2_, no suffix for G) is the radius of M*_i_* or G. Equation (3) expanded as a power series of *ω_i_* and *l_i_* is:

(4) $$\xi_{i}=\frac{1}{2R_{i}}\omega_{i}^{2}+\frac{1}{2R_{i}}l_{i}^{2}+\frac{1}{8R_{i}^{3}}\omega_{i}^{4}+\frac{1}{4R_{i}^{3}}\omega_{i}^{2}l_{i}^{2}+\frac{1}{8R_{i}^{3}}l_{i}^{4}+O(\frac{\omega_{i}^{6}}{R_{i}^{3}}).$$

The angles of incidence θ*_i_* and reflection/diffraction θ*_i_*′ of the principal ray at the vertices of M_i_ (or G) are considered as positive or negative depending on whether the relevant principal ray lies in the first or fourth quadrant of the *x_i_y_i_z_i_* coordinate system. The angles θ and θ′ are related through the grating equation,

(5) $$\sigma (\sin\theta +\sin\theta^{\prime })=m\lambda ,$$

where σ is the effective grating constant obtained by:

(6) $$\sigma \equiv {1/\left(\partial n/\partial \omega \right)}_{\omega =l=0}=\lambda_{0}/n_{10},$$

which can be referred to [26].

## 3. Ray-Tracing Formulas

First, we denote the distances AQ_1_, Q_1_P, PQ_2_, and Q_2_B by *q*_1_, *p*_1_, *q*_2_, and *p*_2_, respectively. According to Namioka’s theory, the application of Fermat’s principle to the light-path function for M_i_,

(7) $$F_{M_{i}}=q_{i}+p_{i},$$

yields the direction cosines (*L*_i_′, *M*_i_′, *N*_i_′) of the reflected ray *p*_i_ in terms of the direction cosines (*L*_i_, *M*_i_, *N*_i_) of the incident ray *q*_i_ and given system parameters:

(8) $$\begin{array}{l} {L^{\prime }}_{i}=L_{i}+\tau_{i},\\ {M^{\prime }}_{i}=M_{i}-\tau_{i}\left(\frac{\partial \xi_{i}}{\partial \omega_{i}}\right),\\ {N^{\prime }}_{i}=N_{i}-\tau_{i}\left(\frac{\partial \xi_{i}}{\partial l_{i}}\right). \end{array}$$

where all the quantities are defined in the *x*_i_*y*_i_*z*_i_ coordinate system. In Equation (8), we have

(9) $$\tau_{i}=\frac{2[-L_{i}+M_{i}(\partial \xi_{i}/\partial \omega_{i})+N_{i}(\partial \xi_{i}/\partial l_{i})]}{1+{\left(\partial \xi_{i}/\partial \omega_{i}\right)}^{2}+{\left(\partial \xi_{i}/\partial l_{i}\right)}^{2}},$$

where *i* = 1, 2 for M_1_ and M_2_. *L*_i_, *M*_i_, and *N*_i_ are obtained from the definition of the direction cosines of the incident ray *q*_i_.

The intersecting point P (*ξ*, *ω*, *l*) is determined by solving simultaneously the equation of the ray Q_1_P in the xyz coordinate system

(10) $$\frac{\xi -{\bar{\xi }}_{1}}{L}=\frac{\omega -{\bar{\omega }}_{1}}{M}=\frac{l-{\bar{l}}_{1}}{N},$$

and Equation (3) with i = 1. In Equation (10) (${\bar{\xi }}_{1}$, ${\bar{\omega }}_{1}$_1_, ${\bar{l}}_{1}$) and (L, M, N) are the coordinates of the point Q_1_ and the direction cosines of the ray Q_1_P, which are both defined in the xyz coordinate system. They are obtained by applying proper coordinate transformations to (ξ_1_, ω_1_, l_1_) and (L_1_′, M_1_′, N_1_′).

Different from the above calculation for M_i_, the application of Fermat’s principle to the light-path function for G,

(11) $$F=p_{1}+q_{2}+nm\lambda ,$$

yields the direction cosines (*L*′, *M*′, *N*′) of the diffracted ray PQ_2_ in terms of the direction cosines (*L*, *M*, *N*) of the incident ray Q_1_P and given system parameters:

(12) $$\begin{array}{l} L^{\prime }=L+\tau ,\\ M^{\prime }=M+m\lambda \left(\frac{\partial n}{\partial \omega }\right)-\tau \left(\frac{\partial \xi }{\partial \omega }\right),\\ N^{\prime }=N+m\lambda \left(\frac{\partial n}{\partial l}\right)-\tau \left(\frac{\partial \xi }{\partial l}\right). \end{array}$$

where all the quantities are defined in the xyz coordinate system. In Equation (12), we have

(13) $$\begin{array}{l} \tau =\frac{1}{\rho }\left(\nu +\sqrt{\nu^{2}-\kappa \rho }\right),\\ \rho =1+{\left(\frac{\partial \xi }{\partial \omega }\right)}^{2}+{\left(\frac{\partial \xi }{\partial l}\right)}^{2},\\ \nu =-L+\left(M+m\lambda \frac{\partial n}{\partial \omega }\right)\frac{\partial \xi }{\partial \omega }+\left(N+m\lambda \frac{\partial n}{\partial l}\right)\frac{\partial \xi }{\partial l},\\ \kappa =2m\lambda \left(M\frac{\partial n}{\partial \omega }+N\frac{\partial n}{\partial l}\right)+{\left(m\lambda \right)}^{2}\left[{\left(\frac{\partial n}{\partial \omega }\right)}^{2}+{\left(\frac{\partial n}{\partial l}\right)}^{2}\right]. \end{array}$$

The intersecting point Q_2_ (*ξ*_2_, *ω*_2_, *l*_2_) is determined by solving simultaneously Equation (3) with *i* = 2 and the equation of ray PQ_2_ in the x_2_y_2_z_2_ coordinate system,

(14) $$\frac{\xi_{2}-\bar{\xi }}{L_{2}}=\frac{\omega_{2}-\bar{\omega }}{M_{2}}=\frac{l_{2}-\bar{l}}{N_{2}},$$

where ($\bar{\xi }$, $\bar{\omega }$, $\bar{l}$) and (L_2_, M_2_, N_2_) are the coordinates of point P and the direction cosines of ray PQ_2_, both defined in the x_2_y_2_z_2_ coordinate system. They are obtained by applying proper coordinate transformations to (ξ, ω, l) and (L′, M′, N′).

The image plane Σ is expressed in the x_2_y_2_z_2_ coordinate system as

(15) $$x_{2}\cos{\theta^{\prime }}_{2}+y_{2}\sin{\theta^{\prime }}_{2}=r_{2}.$$

Then, the intersection B of the reflected ray Q_2_B with the image plane Σ is determined by solving the equation of the ray Q_2_B in the x

_2_y_2_z_2_ coordinate system,

(16) $$\frac{x_{2}-\xi_{2}}{{L^{\prime }}_{2}}=\frac{y_{2}-\omega_{2}}{{M^{\prime }}_{2}}=\frac{z_{2}-l_{2}}{{N^{\prime }}_{2}},$$

from which we obtain:

(17) $$x_{2}=\xi_{2}+p_{2}{L^{\prime }}_{2},\;y_{2}=\omega_{2}+p_{2}{M^{\prime }}_{2},\;z_{2}=l_{2}+p_{2}{N^{\prime }}_{2}.$$

By applying proper coordinate transformations to B (*x*_2_, *y*_2_, *z*_2_), the ray-traced spot B (0, Y, Z) in the XYZ coordinate system is expressed as

(18) $$Y=\left(r_{2}\sin{\theta^{\prime }}_{2}-y_{2}\right)\sec{\theta^{\prime }}_{2},\text{​​​​ }Z=z_{2}.$$

All the above equations presented in this section provide a complete set of ray-tracing formulas.

## 4. Analytic Expression of Spot Diagrams and Aberrations

The imaging characteristics of the three-concentric-element optical system may be analyzed numerically using ray tracing. Although ray tracing provides accurate spot diagrams with comparative ease, it lacks the ability to give explicit analytical expressions for the focal condition and individual aberrations of the system under consideration. According to Namioka’s theory, we express the relationship between the coordinates of a source point and its image by expanding the ray-tracing formulas given in Section 3 into power series of *ω*_1_, *l*_1_, and the coordinates of A_0_ in the X_S_Y_S_Z_S_ system. In this way—although laborious—a third-order aberration theory is developed for the system, which has a high degree of validity.

Taking the expansion of the coordinates of point P as an example, we determine its position in the xyz coordinate system by finding the intersection of ray Q_1_P with the grating blank surface. We express *ω* and *l* in a power series of *ω*_1_^h^*l*_1_^i^z^j^s^k^ (h + i + j + k ≤ 3) under assumptions of:

(19) $$\omega =\sum_{h+i+j+k=1}^{3}A_{hijk}\omega_{1}^{h}l_{1}^{i}z^{j}s^{k},\;l=\sum_{h+i+j+k=1}^{3}B_{hijk}\omega_{1}^{h}l_{1}^{i}z^{j}s^{k}.$$

To determine *ω* and *l*, we derive first the direction cosines (L, M, N) as power series of *ω*_1_, *l*_1_, s and z by expanding their definitions:

(20) $$L=\frac{\xi -{\bar{\xi }}_{1}}{p_{1}},\;M=\frac{\omega -{\bar{\omega }}_{1}}{p_{1}},\;N=\frac{l-{\bar{l}}_{1}}{p_{1}},$$

as

(21) $$\begin{array}{l} L=\sum_{h+i+j+k=1}^{3}{\left(H_{hijk}\right)}_{L}\omega_{1}^{h}l_{1}^{i}z^{j}s^{k},\;\\ M=\sum_{h+i+j+k=1}^{3}{\left(H_{hijk}\right)}_{M}\omega_{1}^{h}l_{1}^{i}z^{j}s^{k},\;\\ N=\sum_{h+i+j+k=1}^{3}{\left(H_{hijk}\right)}_{N}\omega_{1}^{h}l_{1}^{i}z^{j}s^{k}. \end{array}$$

where coefficients (*H_hijk_*)_L_, (*H_hijk_*)_M_, and (*H_hijk_*)_N_ are functions of R_1_, r, θ_1_, and θ only.

Next, we adopt another approach to expand the direction cosines of the ray Q_1_P in terms of *L*_1_′, *M*_1_′, and *N*_1_′:

(22) $$\begin{array}{l} L^{\prime }=\sum_{h+i+j+k=1}^{3}{\left(H_{hijk}\right)}^{\prime }{}_{L}\omega_{1}^{h}l_{1}^{i}z^{j}s^{k},\;\\ M^{\prime }=\sum_{h+i+j+k=1}^{3}{\left(H_{hijk}\right)}^{\prime }{}_{M}\omega_{1}^{h}l_{1}^{i}z^{j}s^{k},\;\\ N^{\prime }=\sum_{h+i+j+k=1}^{3}{\left(H_{hijk}\right)}^{\prime }{}_{N}\omega_{1}^{h}l_{1}^{i}z^{j}s^{k}. \end{array}$$

where the coefficients (*H_hijk_*)_L_′, (*H_hijk_*)_M_′, and (*H_hijk_*)_N_′ are functions of R_1_, r_1_, θ_1_, and θ only. We obtain coefficients *A_hijk_* and *B_hijk_* by equating coefficients (*H_hijk_*)_L_, (*H_hijk_*)_M_, and (*H_hijk_*)_N_ of Equation (21) to the corresponding ones, (*H_hijk_*)_L_′, (*H_hijk_*)_M_′, and (*H_hijk_*)_N_′ of Equation (22), which determines the coefficients A_hijk_ and B_hijk_ uniquely. Therefore, the coordinates of the intersecting point P in terms of *ω*_1_, *l*_1_, s and z are

(23) $$\begin{array}{l} \omega =A_{1000}\omega_{1}+A_{0001}s+A_{2000}\omega_{1}^{2}+A_{1001}\omega_{1}s+A_{0002}s^{2}+A_{0200}l_{1}^{2}\\ \;+A_{0110}l_{1}z+A_{0020}z^{2}+A_{3000}\omega_{1}^{3}+A_{2001}\omega_{1}^{2}s+A_{1200}\omega_{1}l_{1}^{2}+A_{1110}\omega_{1}l_{1}z\\ \;+A_{1020}\omega_{1}z^{2}+A_{1002}\omega_{1}s^{2}+A_{0201}l_{1}^{2}s+A_{0111}l_{1}zs+A_{0021}z^{2}s+A_{0003}s^{3}, \end{array}$$

(24) $$\begin{array}{l} l=B_{0100}l_{1}+B_{0010}z+B_{1100}\omega_{1}l_{1}+B_{1010}\omega_{1}z+B_{0101}l_{1}s+B_{0011}zs+B_{2100}\omega_{1}^{2}l_{1}\\ \;+B_{2010}\omega_{1}^{2}z+B_{1101}\omega_{1}l_{1}s+B_{1011}\omega_{1}zs+B_{0300}l_{1}^{3}+B_{0210}l_{1}^{2}z+B_{0120}l_{1}z^{2}\\ \;+B_{0102}l_{1}s^{2}+B_{0012}zs^{2}+B_{0030}z^{3}. \end{array}$$

Explicit expressions of *A_hijk_* and *B_hijk_* that are applicable to spherical mirror M_1_ are given in [24].

This expansion method for the coordinates of P is used as well to derive power series expressions of the coordinates of Q_2_ and those of B in the x_2_y_2_z_2_ and XYZ coordinate system, respectively. Then, the coordinates (0, Y, Z) of the ray-traced spot B formed in the image plane Σ, which are determined through Equations (15) to (18), are finally expressed as power series in *ω*_1_^h^*l*_1_^i^z^j^s^k^,

(25) $$\begin{array}{l} Y=E_{1000}\omega_{1}+E_{0001}s+E_{2000}\omega_{1}^{2}+E_{1001}\omega_{1}s+E_{0002}s^{2}+E_{0200}l_{1}^{2}+E_{0110}l_{1}z\\ \;+E_{0020}z^{2}+E_{3000}\omega_{1}^{3}+E_{2001}\omega_{1}^{2}s+E_{100}\omega_{1}l_{1}^{2}+E_{1110}\omega_{1}l_{1}z+E_{1020}\omega_{1}z^{2}\\ \;+E_{1002}\omega_{1}s^{2}+E_{0201}l_{1}^{2}s+E_{0111}l_{1}zs+E_{0021}z^{2}s+E_{0003}s^{3}+O_{E}({\kappa^{\prime }}^{4}), \end{array}$$

(26) $$\begin{array}{l} Z=F_{0100}l_{1}+F_{0010}z+F_{1100}\omega_{1}l_{1}+F_{1010}\omega_{1}z+F_{0101}l_{1}s+F_{0011}zs+F_{2100}\omega_{1}^{2}l_{1}\\ \;+F_{2010}\omega_{1}^{2}z+F_{1101}\omega_{1}l_{1}s+F_{1011}\omega_{1}zs+F_{0300}l_{1}^{3}+F_{0210}l_{1}^{2}z+F_{0120}l_{1}z^{2}\\ \;+F_{0102}l_{1}s^{2}+F_{0012}zs^{2}+F_{0030}z^{3}+O_{F}({\kappa^{\prime }}^{4}). \end{array}$$

These two equations are the spot-diagram formulas for the three-concentric optical system. κ′^4^ represent the aberration terms *ω*_1_^h^*l*_1_^i^z^j^s^k^ with h + i + j + k ≥ 4. *O*_E_ and *O*_F_ denote the higher-order terms in the aberration coefficients. The coefficients *E_hijk_* and *F_hijk_* are the aberration coefficients, and we express them in terms of *A_hijk_*, *A_hijk_′*, *A_hijk_″*, *B_hijk_*, *B_hijk_′*, and *B_hijk_″* in Appendix A and Appendix B. Here *A_hijk_′*, *A_hijk_″*, *B_hijk_′*, and *B_hijk_″* are defined as:

(27) $$\begin{array}{l} {A^{\prime }}_{hijk}=A_{hijk}|{}_{r_{1}\to r,r\to r^{\prime },\theta \to \theta_{2},R\to R_{2},\varepsilon_{1}\to \varepsilon },\\ {B^{\prime }}_{hijk}=B_{hijk}|{}_{r_{1}\to r,r\to r^{\prime },\theta \to \theta_{2},R\to R_{2},\varepsilon_{1}\to \varepsilon }, \end{array}$$

(28) $$\begin{array}{l} {A^{\prime \prime }}_{hijk}={A^{\prime }}_{hijk}|{}_{r\to r^{\prime },r^{\prime }\to r_{2},\theta_{2}\to 0,R_{2}\to \infty ,\varepsilon \to \varepsilon_{2}},\\ {B^{\prime \prime }}_{hijk}={B^{\prime }}_{hijk}|{}_{r\to r^{\prime },r^{\prime }\to r_{2},\theta_{2}\to 0,R_{2}\to \infty ,\varepsilon \to \varepsilon_{2}}, \end{array}$$

where *r*_1_ → *r*, for example, indicates replacement of *r*_1_ in *A_hijk_* and *B_hijk_* by *r*. In Equation (27), *ε*_1_ represents all the parameters with a subscript 1, except *r*_1,_ in coefficients *A_hijk_* and *B_hijk_*, and *ε* stands for the corresponding parameters with no subscript in *A_hijk_′* and *B_hijk_′*.

## 5. Analysis of Focal Conditions and Aberrations

For demonstrating various aberrations curves in the next section and evaluating the spot-diagram formulas, we adopt a well-designed and optimized Offner imaging spectrometer as a model. The values of the specific parameters are listed in Table 1; here, the signs of the values are determined by the sign convention.

### 5.1. Focal Conditions

When the first-order aberration coefficients *E*_1000_ and *F*_0100_ are made zero, a configuration with the appropriate instrument parameters is obtained. In such a configuration, the paraxial rays in the meridional or sagittal plane are brought into focus, greatly reducing the aberration of the system. The conditions *E*_1000_ = 0 and *F*_0100_ = 0 give the meridional and sagittal focal curves, respectively.

#### 5.1.1. Meridional Focal Condition

The meridional focal condition *E*_1000_ = *C*_1000_ *A*_1000_ + *C*_0001_ = 0 is expressed as

(29) $$2{\left(F_{1}\right)}_{20}{\left(F*\right)}_{20}=\frac{\cos^{2}{\theta^{\prime }}_{1}\cos^{2}\theta }{r^{2}},\;2{\left(F\right)}_{20}{\left(F_{2}*\right)}_{20}=\frac{\cos^{2}\theta^{\prime }\cos^{2}\theta_{2}}{{r^{\prime }}^{2}},$$

where (F∗)_20_ is the value of (*F*)_20_ at *r*′ = (*r*″)_M_, and ()_20_ is the value of ($F_{2}*$)_20_ at *r*_2_ = (*r*_2_′)_M_. The focal distances *r*′ = (*r*″)_M_ and *r*_2_ = (*r*_2_′)_M_ that satisfy Equation (29) are called the meridional focal distances of *G* and M_2_ respectively. (*F*_1_)_20_, (*F*)_20_, and (*F*_2_)_20_ are defined as

(30) $$\begin{array}{l} {\left(F_{1}\right)}_{20}=\frac{\cos^{2}\theta_{1}}{2r_{1}}+\frac{\cos^{2}{\theta^{\prime }}_{1}}{2r}-\frac{2\cos\theta_{1}}{R_{1}},\\ {\left(F\right)}_{20}=\frac{\cos^{2}\theta }{2r}+\frac{\cos^{2}\theta^{\prime }}{2r^{\prime }}-\frac{\cos\theta +\cos\theta^{\prime }}{R}+{\left(n\right)}_{20}\Pi ,\\ {\left(F_{2}\right)}_{20}=\frac{\cos^{2}\theta_{2}}{2r^{\prime }}+\frac{\cos^{2}{\theta^{\prime }}_{2}}{2r_{2}}-\frac{2\cos\theta_{2}}{R_{2}} \end{array}$$

Hence, Equation (29) reduces to:

(31) $$2{\left(F_{1}\right)}_{20}=\frac{\cos^{2}{\theta^{\prime }}_{1}}{r}-\frac{\cos^{2}{\theta^{\prime }}_{1}}{{\left({r^{\prime }}_{1}\right)}_{M}},\;{\left(F\right)}_{20}=\frac{\cos^{2}\theta }{r}-\frac{\cos^{2}\theta }{{\left(r\right)}_{M}},$$

(32) $${\left(F\right)}_{20}=\frac{\cos^{2}\theta^{\prime }}{r^{\prime }}-\frac{\cos^{2}\theta^{\prime }}{{\left(r^{\prime \prime }\right)}_{M}},\;2{\left(F_{2}\right)}_{20}=\frac{\cos^{2}\theta_{2}}{r^{\prime }}-\frac{\cos^{2}\theta_{2}}{{\left(r_{2}\right)}_{M}},$$

where the meridional focal conditions for M_1_, G, and M_2_ are expressed, separately. In Equation (31), (*r*_1_′)_M_ is the meridional focal distance of M_1_, giving the object distance of G in the meridional plane as (*r*)_M_ = *r* − (*r*_1_′)_M_. Similarly, the object distance of M_2_ in the meridional plane is obtained from Equation (32) as (*r*_2_)_M_ = *r*′ − (*r*″)_M_. We then obtain the meridional focal distance of the Offner optical system by solving Equations (30) to (32).

#### 5.1.2. Sagittal Focal Condition

We present the sagittal condition *F*_0100_ = *D*_0010_ + *D*_0100_ *B*_0100_ = 0 as

(33) $$2{\left(F_{1}\right)}_{02}{\left(F^{*}\right)}_{02}=\frac{1}{r^{2}},\;2{\left(F\right)}_{02}{\left(F_{2}{}^{*}\right)}_{02}=\frac{1}{{r^{\prime }}^{2}},$$

where (F^*^)_02_ is the value of (*F*)_02_ at *r*′ = (*r*″)_S_, and (*F*_2_^*^)_02_ is the value of (*F*_2_)_02_ at *r*_2_ = (*r*_2_′)_S_. The focal distances *r*′ = (*r*″)_S_ and *r*_2_ = (*r*_2_′)_S_ that satisfy Equation (33), are called the sagittal focal distances of G and M_2_, respectively. (*F*_1_)_02_, (*F*)_02_, and (*F*_2_)_02_ are defined by

(34) $$\begin{array}{l} {\left(F_{1}\right)}_{02}=\frac{1}{2r_{1}}+\frac{1}{2r}-\frac{2\cos\theta_{1}}{R_{1}},\\ {\left(F\right)}_{02}=\frac{1}{2r}+\frac{1}{2r^{\prime }}-\frac{\cos\theta +\cos\theta^{\prime }}{R}+{\left(n\right)}_{02}\Pi ,\\ {\left(F_{2}\right)}_{02}=\frac{1}{2r^{\prime }}+\frac{1}{2r_{2}}-\frac{2\cos\theta_{2}}{R_{2}}. \end{array}$$

Similar to obtaining the meridional focus, we resolve Equation (33) into:

(35) $$2{\left(F_{1}\right)}_{02}=\frac{1}{r}-\frac{1}{{\left({r^{\prime }}_{1}\right)}_{S}},\;{\left(F\right)}_{02}=\frac{1}{r}-\frac{1}{{\left(r\right)}_{S}},$$

(36) $${\left(F\right)}_{02}=\frac{1}{r^{\prime }}-\frac{1}{{\left(r^{\prime \prime }\right)}_{S}},\;2{\left(F_{2}\right)}_{02}=\frac{1}{r^{\prime }}-\frac{1}{{\left(r_{2}\right)}_{S}},$$

which represent the sagittal focal conditions for the three elements of the system. Likewise, we obtain the object distances of G and M_2_ in the sagittal plane in the form (*r*)_S_ = *r* − (*r*_1_′)_S_ and (*r*_2_)_S_ = *r*′ − (*r*″)_S_. Here (*r*_1_′)_S_ in Equation (35) is the sagittal focal distance of G. Therefore, the sagittal focal distance of the system is given by solving Equations (34) and (35).

For a real point source and a real image, the system shown in Figure 1 is capable of making the tangential and sagittal focal points to coincide, yielding non-astigmatic image when (*r*_2_′)_M_ = (*r*_2_′)_S_ is satisfied. Failure to meet the condition leads to the astigmatic aberration.

### 5.2. Aberration Analysis

Next, we introduce the polar coordinates

(37) $$\omega_{1}=r_{p}\cos\alpha ,\;l_{1}=r_{p}\sin\alpha ,$$

in the entrance pupil centered at the vertex O_1_ of M_1_.

#### 5.2.1. Spherical Aberration

In the ray-tracing formulas, spherical aberration is described by

(38) $$Y_{sph}=E_{3000}\omega_{1}^{3}+E_{1200}\omega_{1}l_{1}^{2},\;Z_{sph}=F_{0300}l_{1}^{3}+F_{2100}\omega_{1}^{2}l_{1},$$

which can be changed into:

(39) $$Y_{sph}=r_{p}(E_{3000}\cos^{2}\alpha +E_{1200}\sin^{2}\alpha )\cos\alpha ,\;Z_{sph}=r_{p}(E_{3000}\sin^{2}\alpha +E_{1200}\cos^{2}\alpha )\sin\alpha .$$

**Figure 2:**
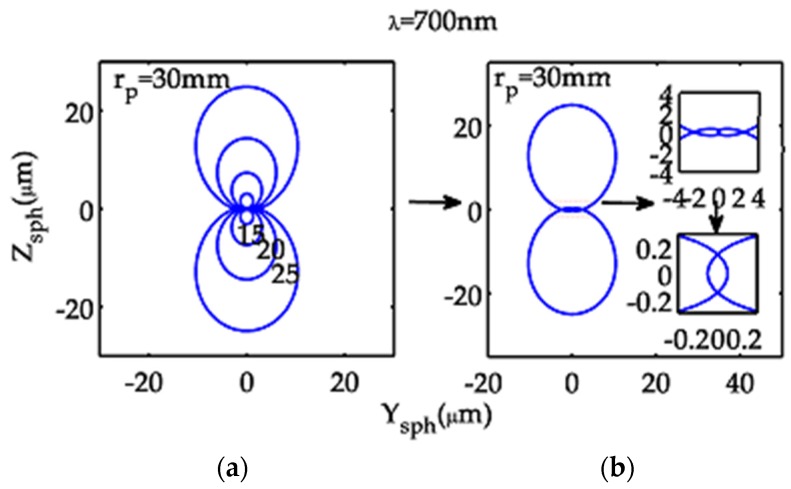
Spherical aberration curves for λ = 700 nm of the model optical system in the meridional focal plane: (**a**) with *r*_p_ = 30, 25, 20, 15 mm and (**b**) with *r*_p_ = 30 mm (each inset is an enlargement of a central portion of the curve).

The spherical aberration curves of the model optical system for the center wavelength (Figure 2) are more complicated than common circular patterns of a centered lens system.

The spherical aberration curve is a circle of *r*_p_^3^*E*_3000_ only when *E*_3000_ = *E*_1200_ = *F*_0300_ = *F*_2100_ is met. This condition is satisfied by an axially symmetric centered Offner optical system, which is the same as both a single mirror and a centered double-mirror system, yielding a concentric circular pattern for various values of *r*_p_.

#### 5.2.2. Coma

The coma of the concentric Offner optical system under consideration is expressed by:

(40) $$\begin{array}{l} Y_{coma}=E_{2000}\omega_{1}^{2}+E_{2010}\omega_{1}^{2}z+E_{0210}l_{1}^{2}z+E_{1101}\omega_{1}l_{1}s,\;\\ Z_{coma}=F_{1100}\omega_{1}l_{1}+F_{2001}\omega_{1}^{2}s+F_{1110}\omega_{1}l_{1}z+F_{0201}l_{1}^{2}s \end{array}$$

Substitution of Equation (37) into Equation (40) yields:

(41) $$\begin{array}{l} a{\left\{\frac{2Y_{coma}}{r^{2}}-\left[E_{2000}+s(E_{2001}+E_{0201})\right]\right\}}^{2}+b{\left\{\frac{2Z_{coma}}{r^{2}}-z(F_{2010}+F_{0210})\right\}}^{2}\\ -2h\left\{\frac{2Y_{coma}}{r^{2}}-\left[E_{2000}+s(E_{2001}+E_{0201})\right]\right\}\left\{\frac{2Z_{coma}}{r^{2}}-z(F_{2010}+F_{0210})\right\}=c^{2}, \end{array}$$

where

(42) $$\begin{array}{l} a={\left(F_{1100}+sF_{1101}\right)}^{2}+z^{2}{\left(F_{2010}-F_{0210}\right)}^{2},\\ b={\left[E_{2000}+s(E_{2001}-E_{0201})\right]}^{2}+z^{2}E_{1110}^{2},\\ c=(F_{1100}+sF_{1101})\left[E_{2000}+s(E_{2001}-E_{0201})\right]-z^{2}E_{1110}(F_{2010}-F_{0210}),\\ h=z\left\{E_{1110}(F_{1100}+sF_{1101})+(F_{2010}-F_{0210})\left[E_{2000}+s(E_{2001}-E_{0201})\right]\right\}. \end{array}$$

**Figure 3:**
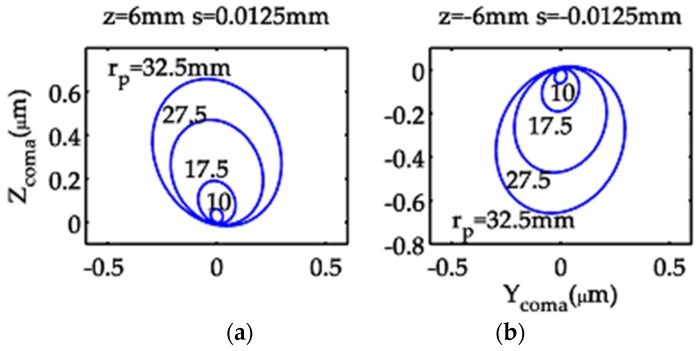
Coma curves of the model optical system in the meridional focal plane, illustrating changes in pattern with *r*_p_, for settings (**a**) z = 6 mm and s = 0.0125 mm and (**b**) z = −6 mm and s = −0.0125 mm.

With Equation (41) describing an ellipse, the model optical system produces elliptical patterns for different values of *r*_p_ (Figure 3).

#### 5.2.3. Astigmatism

Astigmatism is an image defect caused by two mutually perpendicular line images, one at (*r*_2_′)_M_ and the other at (*r*_2_′)_S_. Astigmatism of the concentric Offner optical system is represented by:

(43) $$\begin{array}{l} Y_{ast}=E_{0001}s+E_{0200}l_{1}^{2}+E_{0110}l_{1}z+E_{0020}z^{2},\;\\ Z_{ast}=F_{0100}l_{1}+F_{0010}z. \end{array}$$

which transforms to

(44) $$Y_{ast}=E_{0200}{\left[\frac{Z_{ast}}{F_{0100}}+\left(\frac{E_{0110}}{2E_{0200}}-\frac{F_{0010}}{F_{0100}}\right)z\right]}^{2}+\left(E_{0020}-\frac{E_{0110}^{2}}{4E_{0200}}\right)z^{2}+E_{0001}s.$$

**Figure 4:**
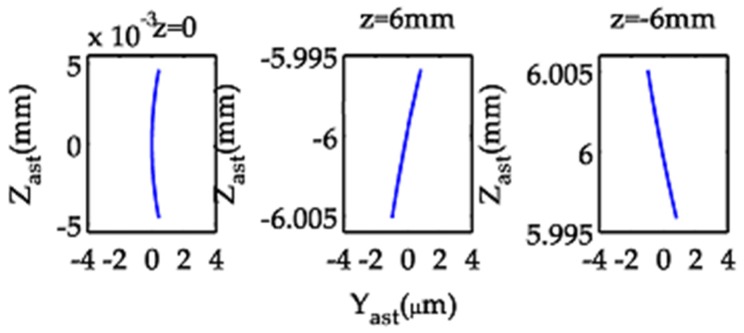
Astigmatic curves of the model optical system in the meridional focal plane.

The astigmatic curves obtained from the model optical system appear as crescent-shaped patterns (Figure 4).

#### 5.2.4. Distortion

**Figure 5:**
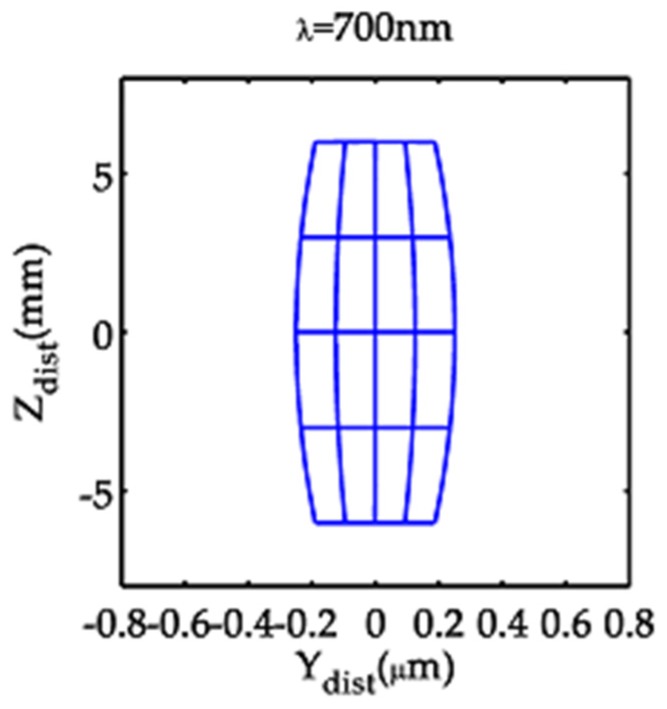
Distortion patterns of the model optical system for a mesh-like source of dimension 25 μm (s) × 12 mm (z) with line separations of Δs = 6.25 μm and Δz = 3 mm.

Distortion is the deviation between the actual image height and the ideal image height of the chief ray originating from a source point (0, s, z) and passing through the vertex O_1_. In the ray-tracing formulas, the distortion is expressed as

(45) $$\begin{array}{l} Y_{dist}=E_{0001}s+E_{0020}z^{2}+E_{0002}s^{2}+E_{0021}z^{2}s+E_{0003}s^{3},\\ Z_{dist}=F_{0010}z+F_{0011}zs+F_{0030}z^{3}+F_{0012}zs^{2}. \end{array}$$

which manifests as a barrel-like structure from the model optical system (Figure 5). Because of the use of a very narrow slit illuminant, the distortions are visible in the Z direction and not in the Y direction.

## 6. Analysis of Diagram and Discussion

In Section 5, the evaluation of individual aberrations in the model optical system was presented using aberration curves, facilitating a better understanding of the imaging properties of the Offner system. More importantly, such an evaluation method helps to design and optimize the Offner system with an aberration-correction convex grating for different requirements.

However, before adopting the spot diagram formulas (25) and (26) in the design of an Offner system and its grating, the equations need to be critically evaluated in a comparison with exact ray-tracing. Here we compare spot diagrams computed from Equations (25) and (26) with those determined by the exact ray tracing using ZEMAX configured with a model Offner system equipped with a holographic convex grating.

**Figure 6:**
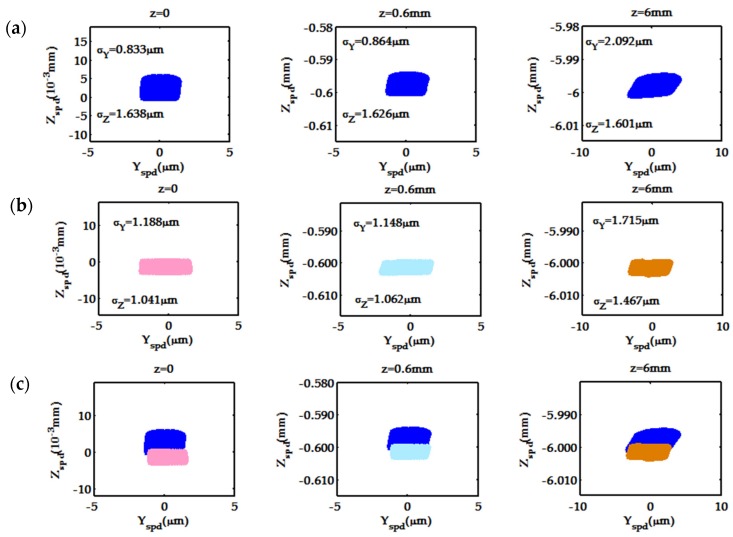
Spot diagrams constructed for the model optical system at λ = 700 nm with s = 0 from: (**a**) formulas (25) and (26); (**b**) ray tracing using ZEMAX; and (**c**) contrasted by overlaying (**a**) with (**b**).

**Figure 7:**
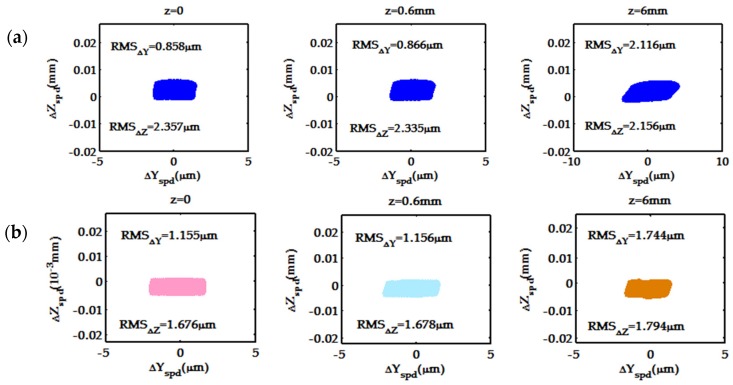
ΔY - ΔZ plots constructed for the model optical system at λ = 700 nm. (**a**) deviations, ΔY - ΔZ, of individual spots in Figure 6a from the corresponding ideal image points (0,0,0), (0,0,0.6) and (0,0,6); (**b**) deviations, ΔY - ΔZ, of individual spots in Figure 6b from the corresponding ideal image points (0,0,0), (0,0,0.6) and (0,0,6)7. Conclusions.

Because of the large z value and a relatively small *s* value in the model system, a portion of the spot diagram was constructed for various fields where we set z = 0, 0.6, and 6 mm with s = 0 without loss of generality. All the diagrams in Figure 6 and Figure 7 were constructed by generating 20000 rays of wavelength 700 nm covering the whole field of view. Spot diagrams in (a) and (b) were constructed for the selected point source presented in Figure 6, using the spot-diagram formulas and by ray tracing using ZEMAX, respectively. Clearly, the spot diagrams in (a) and (b) are similar in shape, but there are some deviations in size and position—especially in the Z direction; see Figure 6c.

The standard deviations *σ*_Y_ and *σ*_Z_ of the spots in the Y and Z directions (Figure 6a,b) illustrate the similarity in spot shape. The difference between the standard deviations of corresponding individual spots is smaller than 0.4 μm in the Y direction and 0.65 μm in the Z direction. Nearly the same dispersion tendency is seen depending on the system aberrations for the spot diagrams generated by both the present theoretical model and the simulation model of ZEMAX.

Figure 7a shows the deviations of individual spots obtained by the spot-diagram formulas from the corresponding ideal image points (0,0,0), (0,0,0.6) and (0,0,6). Figure 7b shows the deviations of individual spots generated by ray tracing using ZEMAX from the corresponding ideal image points. The deviations between ideal image points and spots from a simulation model (such as the theoretical simulation model or the ZEMAX simulation model) depend on both the system aberrations and model errors. The root-mean-squares RMS_ΔY_ and RMS_ΔZ_ of the deviations are given in the respective diagrams. Here, distinctions between the individual corresponding spots in Figure 7a,b—both in size and position—are mainly determined by different model errors. However, the difference between RMS_ΔY_ and RMS_ΔZ_ of the spots in Figure 7a,b is smaller than 0.4 μm in the Y direction and 0.7 μm in the Z direction. Therefore, the present theoretical model is similarly as useful as the ZEMAX model in designing and optimizing the Offner optical system. Certainly, supplemented by the fourth- and higher-order aberration terms into the spot diagram formulas (25) and (26), more exact theoretical model may be developed.

## 7. Conclusions

In this paper, a more practical method is adopted, comparing the theoretical simulation model with the simulation model provided by the commercial software ZEMAX, and offers great practicality.

A third-order aberration geometric theory was developed for tracing rays through the Offner imaging spectrometer comprising an extended source, two concave mirrors, a convex diffraction grating, and an image plane based on Fermat’s principle. The proposed theory provides analytic formulas for individual aberrations and spot diagrams. Following on from Namioka’s work, aberrations were analyzed and certain aberration curves were illustrated for a corresponding model optical system. The validity of the theoretical model was evaluated in a comparison with a simulation model provided by the commercial software ZEMAX and that of an actual model optical system. The results indicate the proposed theoretical model has great utility and practicality. 

## Figures and Tables

**Table 1:** Parameters of the model Offner imaging spectrometer.

Parameter	Value
Spectral range/nm	380–900
Radius of M_1_/mm	220
Radius of G/mm	112.2
Radius of M_2_/mm	216.85
Dimension of slit/mm^2^	0.025 × 12
Aperture of M_1_/mm^2^	65 × 65
Aperture of G/mm^2^	30 × 30
Constant of G/mm^−1^	0.01
Diffraction order of G	−1
∠O_1_OO_2_	50.66°

## Appendix A. Aberration Coefficients *C_hijk_* and *D_hijk_*

(A1) $$C_{1000}=A_{1000}{}^{\prime }A_{1000}{}^{\prime \prime }+A_{0001}{}^{\prime \prime }\cos\theta^{\prime },$$

(A2) $$C_{0001}=A_{0001}{}^{\prime }A_{1000}{}^{\prime \prime }\cos\theta_{1}{}^{\prime },$$

(A3) $$\begin{array}{l} C_{2000}=A_{2000}{}^{\prime }A_{1000}{}^{\prime \prime }+A_{1000}{{}^{\prime }}^{2}A_{2000}{}^{\prime \prime }+A_{1000}{}^{\prime }A_{1001}{}^{\prime \prime }\cos\theta^{\prime }+A_{0002}{}^{\prime \prime }\cos^{2}\theta^{\prime }-A_{0001}{}^{\prime \prime }\sin\theta^{\prime }\\ \;\times \left(\frac{1}{2R}-\frac{\cos\theta^{\prime }}{r^{\prime }}-\frac{A_{1000}{}^{\prime }\cos\theta_{2}}{r^{\prime }}\right), \end{array}$$

(A4) $$\begin{array}{l} C_{1001}=A_{1001}{}^{\prime }A_{1000}{}^{\prime \prime }\cos\theta_{1}{}^{\prime }+2A_{0001}{}^{\prime }A_{1000}{}^{\prime }A_{2000}{}^{\prime \prime }\cos\theta_{1}{}^{\prime }+A_{0001}{}^{\prime }A_{1001}{}^{\prime \prime }\cos\theta_{1}{}^{\prime }\cos\theta^{\prime }\\ \;+\frac{A_{0001}{}^{\prime }A_{1000}{}^{\prime \prime }\sin\theta_{1}{}^{\prime }\cos\theta }{r}+\frac{A_{0001}{}^{\prime }A_{0001}{}^{\prime \prime }\cos\theta_{1}{}^{\prime }\sin\theta^{\prime }\cos\theta_{2}}{r^{\prime }}, \end{array}$$

(A5) $$C_{0200}=A_{0020}{}^{\prime \prime }+A_{0200}{}^{\prime }A_{1000}{}^{\prime \prime }+A_{0110}{}^{\prime \prime }B_{0100}{}^{\prime }+A_{0200}{}^{\prime \prime }B_{0100}{{}^{\prime }}^{2}-\frac{A_{0001}{}^{\prime \prime }\sin\theta^{\prime }}{2R},$$

(A6) $$C_{0110}=A_{0110}{}^{\prime }A_{1000}{}^{\prime \prime }+A_{0110}{}^{\prime \prime }B_{0010}{}^{\prime }+2A_{0200}{}^{\prime \prime }B_{0010}{}^{\prime }B_{0100}{}^{\prime },$$

(A7) $$C_{0020}=A_{0020}{}^{\prime }A_{1000}{}^{\prime \prime }+A_{0200}{}^{\prime \prime }B_{0010}{{}^{\prime }}^{2}-\frac{A_{0001}{}^{\prime }A_{1000}{}^{\prime \prime }\sin\theta_{1}{}^{\prime }}{2R_{1}},$$

(A8) $$C_{0002}=A_{0002}{}^{\prime }A_{1000}{}^{\prime \prime }\cos^{2}\theta_{1}{}^{\prime }+A_{0001}{}^{\prime }A_{1000}{}^{\prime \prime }\left(\frac{\sin\theta_{1}{}^{\prime }\cos\theta_{1}{}^{\prime }}{r}-\frac{\sin\theta_{1}{}^{\prime }}{2R_{1}}\right)+A_{0001}{{}^{\prime }}^{2}A_{2000}{}^{\prime \prime }\cos^{2}\theta_{1}{}^{\prime },$$

(A9) $$\begin{array}{l} C_{3000}=A_{3000}{}^{\prime }A_{1000}{}^{\prime \prime }+2A_{1000}{}^{\prime }A_{2000}{}^{\prime }A_{2000}{}^{\prime \prime }+A_{1000}{{}^{\prime }}^{3}A_{3000}{}^{\prime \prime }+A_{2000}{}^{\prime }A_{1001}{}^{\prime \prime }\cos\theta^{\prime }\\ \;+A_{1000}{{}^{\prime }}^{2}A_{2001}{}^{\prime \prime }\cos\theta^{\prime }+A_{1000}{}^{\prime }A_{1002}{}^{\prime \prime }\cos^{2}\theta^{\prime }+A_{0003}{}^{\prime \prime }\cos^{3}\theta^{\prime }-\left(\frac{1}{2R}-\frac{\cos\theta^{\prime }}{r^{\prime }}\right)\\ \;\times (\frac{r_{3}A_{1000}{}^{\prime }\cos\theta^{\prime }\cos^{2}\theta_{2}\sec\theta_{2}{}^{\prime }}{r^{\prime }{}^{2}}+A_{1000}{}^{\prime }A_{1001}{}^{\prime \prime }\sin\theta^{\prime }+2A_{0002}{}^{\prime \prime }\sin\theta^{\prime }\cos\theta^{\prime }\\ \;+\frac{A_{0001}{}^{\prime \prime }\sin^{2}\theta^{\prime }}{r^{\prime }})-\frac{A_{1000}{{}^{\prime }}^{2}A_{0001}{}^{\prime \prime }\sin\theta^{\prime }\sin\theta_{2}}{2R_{2}r^{\prime }}+\frac{A_{0001}{}^{\prime \prime }\cos^{2}\theta^{\prime }}{2Rr^{\prime }}+\frac{\sin\theta^{\prime }\cos\theta_{2}}{r^{\prime }}\\ \;\times \left(A_{2000}{}^{\prime }A_{0001}{}^{\prime \prime }+A_{1000}{{}^{\prime }}^{2}A_{1001}{}^{\prime \prime }\right)-\frac{r_{2}A_{1000}{}^{\prime }}{r^{\text{′}3}}(\cos^{2}\theta_{2}\sec\theta_{2}{}^{\prime }+\sin\theta^{\prime }\cos\theta^{\prime }\cos^{3}\theta_{2}\\ \;\times \sec^{2}\theta_{2}{}^{\prime }\tan\theta_{2}{}^{\prime })+\frac{A_{1000}{}^{\prime }\sin\theta^{\prime }\sin\theta_{2}\cos\theta_{2}}{r^{\prime }{}^{2}}\left(A_{1000}{}^{\prime }A_{0001}{}^{\prime \prime }-\frac{2r_{2}\cos\theta^{\prime }\sec\theta_{2}{}^{\prime }}{r^{\prime }}\right), \end{array}$$

(A10) $$\begin{array}{l} C_{1110}=A_{1110}{}^{\prime }A_{1000}{}^{\prime \prime }+2A_{0110}{}^{\prime }A_{1000}{}^{\prime }A_{2000}{}^{\prime \prime }+A_{1000}{}^{\prime }A_{1110}{}^{\prime \prime }B_{0010}{}^{\prime }+2A_{1000}{}^{\prime }A_{1200}{}^{\prime \prime }B_{0010}{}^{\prime }B_{0100}{}^{\prime }\\ \text{ ​​​ }+A_{0110}{}^{\prime \prime }B_{1010}{}^{\prime }+2A_{0200}{}^{\prime \prime }B_{0100}{}^{\prime }B_{1010}{}^{\prime }+2A_{0200}{}^{\prime \prime }B_{0010}{}^{\prime }B_{1100}{}^{\prime }+A_{0110}{}^{\prime }A_{1001}{}^{\prime \prime }\cos\theta^{\prime }\\ \;+A_{0111}{}^{\prime \prime }B_{0010}{}^{\prime }\cos\theta^{\prime }+2A_{0201}{}^{\prime \prime }B_{0010}{}^{\prime }B_{0100}{}^{\prime }\cos\theta^{\prime }+\frac{A_{0110}{}^{\prime }A_{0001}{}^{\prime \prime }\sin\theta^{\prime }\cos\theta_{2}}{r^{\prime }}\\ \;-\frac{B_{0010}{}^{\prime }\sin\theta^{\prime }}{r^{\prime }}\left(2A_{0020}{}^{\prime \prime }-A_{0110}{}^{\prime \prime }+2A_{0110}{}^{\prime \prime }B_{0100}{}^{\prime }+\frac{A_{0001}{}^{\prime \prime }B_{0100}{}^{\prime }\sin\theta_{2}}{R_{2}}\right), \end{array}$$

(A11) $$\begin{array}{l} C_{2001}=A_{2001}{}^{\prime }A_{1000}{}^{\prime \prime }\cos\theta_{1}{}^{\prime }+2A_{1000}{}^{\prime }A_{1001}{}^{\prime }A_{2000}{}^{\prime \prime }\cos\theta_{1}{}^{\prime }+2A_{0001}{}^{\prime }A_{2000}{}^{\prime }A_{2000}{}^{\prime \prime }\cos\theta_{1}{}^{\prime }\\ \;+3A_{0001}{}^{\prime }A_{1000}{{}^{\prime }}^{2}A_{3000}{}^{\prime \prime }\cos\theta_{1}{}^{\prime }+A_{1001}{}^{\prime }A_{1001}{}^{\prime \prime }\cos\theta_{1}{}^{\prime }\cos\theta^{\prime }+2A_{0001}{}^{\prime }A_{1000}{}^{\prime }A_{2001}{}^{\prime \prime }\\ \;\times \cos\theta_{1}{}^{\prime }\cos\theta^{\prime }+\frac{A_{0001}{}^{\prime }A_{1001}{}^{\prime \prime }\sin\theta_{1}{}^{\prime }\cos\theta \cos\theta^{\prime }}{r}+\frac{A_{1001}{}^{\prime }A_{1000}{}^{\prime \prime }\sin\theta_{1}{}^{\prime }\cos\theta }{r}\\ \;+\frac{2A_{0001}{}^{\prime }A_{1000}{}^{\prime }A_{2000}{}^{\prime \prime }\sin\theta_{1}{}^{\prime }\cos\theta }{r}+\frac{A_{0001}{}^{\prime }A_{1000}{}^{\prime \prime }\sin\theta_{1}{}^{\prime }\sin\theta }{r}\left(\frac{1}{2R}-\frac{\cos\theta }{r}\right)\\ \;-\left(\frac{r_{2}A_{0001}{}^{\prime }\cos\theta_{1}{}^{\prime }\cos\theta^{\prime }\cos^{2}\theta_{2}\sec\theta_{2}{}^{\prime }}{r^{\prime }{}^{2}}+A_{0001}{}^{\prime }A_{1001}{}^{\prime \prime }\cos\theta_{1}{}^{\prime }\sin\theta^{\prime }\right)(\frac{1}{2R}\\ \;-\frac{\cos\theta^{\prime }}{r^{\prime }})-\frac{2A_{0001}{}^{\prime }A_{1000}{}^{\prime }A_{0001}{}^{\prime \prime }\cos\theta_{1}{}^{\prime }\sin\theta^{\prime }\sin\theta_{2}}{r^{\prime }}\times \left(\frac{1}{2R_{2}}-\frac{\cos\theta_{2}}{r^{\prime }}\right)\\ \;+A_{0001}{}^{\prime }A_{1002}{}^{\prime \prime }\cos\theta_{1}{}^{\prime }\cos^{2}\theta^{\prime }+\frac{\sin\theta^{\prime }\cos\theta_{2}}{r^{\prime }}(A_{1001}{}^{\prime }A_{0001}{}^{\prime \prime }\cos\theta_{1}{}^{\prime }+2A_{0001}{}^{\prime }A_{1000}{}^{\prime }\\ \;\times A_{0001}{}^{\prime \prime }\cos\theta_{1}{}^{\prime })+\frac{A_{0001}{}^{\prime }A_{0001}{}^{\prime \prime }\sin\theta_{1}{}^{\prime }\cos\theta \sin\theta^{\prime }\cos\theta_{2}}{rr^{\prime }}-\frac{r_{2}A_{0001}{}^{\prime }\cos\theta_{1}{}^{\prime }}{r^{\prime }{}^{3}}\\ \;\times \cos\theta_{2}\sec\theta_{2}{}^{\prime }\left(\cos\theta_{2}+\sin\theta^{\prime }\cos\theta^{\prime }\left(2\sin\theta_{2}+\cos^{2}\theta_{2}\sec\theta_{2}{}^{\prime }\tan\theta_{2}{}^{\prime }\right)\right), \end{array}$$

(A12) $$\begin{array}{l} C_{1200}=A_{1200}{}^{\prime }A_{1000}{}^{\prime \prime }+A_{1000}{}^{\prime }A_{1020}{}^{\prime \prime }+2A_{0200}{}^{\prime }A_{1000}{}^{\prime }A_{2000}{}^{\prime \prime }+A_{1000}{}^{\prime }A_{1110}{}^{\prime \prime }B_{0100}{}^{\prime }\\ \;+A_{1000}{}^{\prime }A_{1200}{}^{\prime \prime }B_{0100}{{}^{\prime }}^{2}+A_{0110}{}^{\prime \prime }B_{1100}{}^{\prime }+2A_{0200}{}^{\prime \prime }B_{0100}{}^{\prime }B_{1100}{}^{\prime }+A_{0200}{}^{\prime }A_{1001}{}^{\prime \prime }\cos\theta^{\prime }\\ \;+A_{0111}{}^{\prime \prime }B_{0100}{}^{\prime }\cos\theta^{\prime }+A_{0201}{}^{\prime \prime }B_{0100}{{}^{\prime }}^{2}\cos\theta^{\prime }+A_{0021}{}^{\prime \prime }\cos\theta^{\prime }-\frac{A_{0001}{}^{\prime \prime }}{2Rr^{\prime }}\\ \;\times \left(1-A_{1000}{}^{\prime }\cos\theta^{\prime }\cos\theta_{2}-2\cos^{2}\theta^{\prime }\right)+\frac{\sin\theta^{\prime }}{r^{\prime }}(2A_{0020}{}^{\prime \prime }-2A_{0020}{}^{\prime \prime }B_{0100}{}^{\prime }\\ \;+A_{0110}{}^{\prime \prime }B_{0100}{}^{\prime }-A_{0110}{}^{\prime \prime }B_{0100}{{}^{\prime }}^{2}+A_{0200}{}^{\prime }A_{0001}{}^{\prime \prime }\cos\theta_{2})-\frac{\sin\theta^{\prime }}{2R}(A_{1000}{}^{\prime }A_{1001}{}^{\prime \prime }\\ \;+2A_{0002}{}^{\prime \prime }\cos\theta^{\prime })-\frac{A_{0001}{}^{\prime \prime }B_{0100}{{}^{\prime }}^{2}\sin\theta^{\prime }\sin\theta_{2}}{2R_{2}r^{\prime }}, \end{array}$$

(A13) $$\begin{array}{l} C_{1020}=A_{1020}{}^{\prime }A_{1000}{}^{\prime \prime }+2A_{1000}{}^{\prime }A_{0020}{}^{\prime }A_{2000}{}^{\prime \prime }+A_{1000}{}^{\prime }A_{1200}{}^{\prime \prime }B_{0010}{{}^{\prime }}^{2}+2A_{0200}{}^{\prime \prime }B_{0010}{}^{\prime }B_{1010}{}^{\prime }\\ \;+A_{0020}{}^{\prime }A_{1001}{}^{\prime \prime }\cos\theta^{\prime }+A_{0201}{}^{\prime \prime }B_{0010}{{}^{\prime }}^{2}\cos\theta^{\prime }-\frac{A_{0001}{}^{\prime }A_{1001}{}^{\prime \prime }\sin\theta_{1}{}^{\prime }\cos\theta^{\prime }}{2R_{1}}\\ \;-\frac{A_{1000}{}^{\prime }A_{0001}{}^{\prime }A_{2000}{}^{\prime \prime }\sin\theta_{1}{}^{\prime }}{R_{1}}-\frac{A_{1001}{}^{\prime }A_{1000}{}^{\prime \prime }\sin\theta_{1}{}^{\prime }}{2R_{1}}+\frac{A_{0001}{}^{\prime }A_{1000}{}^{\prime \prime }\cos\theta_{1}{}^{\prime }\cos\theta }{2R_{1}r}\\ \;-\frac{\sin\theta^{\prime }}{r^{\prime }}(\frac{A_{0001}{}^{\prime }A_{0001}{}^{\prime \prime }\sin\theta_{1}{}^{\prime }\cos\theta_{2}}{2R_{1}}+A_{0110}{}^{\prime \prime }B_{0010}{{}^{\prime }}^{2}-A_{0020}{}^{\prime }A_{0001}{}^{\prime \prime }\cos\theta_{2}\\ \;+\frac{A_{0001}{}^{\prime }B_{0010}{{}^{\prime }}^{2}\sin\theta_{2}}{2R_{2}}), \end{array}$$

(A14) $$\begin{array}{l} C_{0021}=A_{0021}{}^{\prime }A_{1000}{}^{\prime \prime }\cos\theta_{1}{}^{\prime }+2A_{0001}{}^{\prime }A_{0020}{}^{\prime }A_{2000}{}^{\prime \prime }\cos\theta_{1}{}^{\prime }+A_{0001}{}^{\prime }A_{1200}{}^{\prime \prime }B_{0010}{{}^{\prime }}^{2}\cos\theta_{1}{}^{\prime }\\ \;-\frac{\sin\theta_{1}{}^{\prime }\cos\theta_{1}{}^{\prime }}{R_{1}}\left(A_{0001}{{}^{\prime }}^{2}A_{2000}{}^{\prime \prime }+A_{0002}{}^{\prime }A_{1000}{}^{\prime \prime }\right)-\frac{A_{0001}{}^{\prime }A_{1000}{}^{\prime \prime }}{2R_{1}r}\left(1-2\cos^{2}\theta_{1}{}^{\prime }\right)\\ \;+\frac{2A_{0020}{}^{\prime }A_{1000}{}^{\prime \prime }\sin\theta_{1}{}^{\prime }}{r}+2A_{0200}{}^{\prime \prime }B_{0010}{}^{\prime }\left(-\frac{r_{2}\sin\theta_{1}{}^{\prime }}{r^{2}}+B_{0011}{}^{\prime }\cos\theta_{1}{}^{\prime }\right), \end{array}$$

(A15) $$\begin{array}{l} C_{1002}=A_{1002}{}^{\prime }A_{1000}{}^{\prime \prime }\cos^{2}\theta_{1}{}^{\prime }+A_{0001}{{}^{\prime }}^{2}A_{2001}{}^{\prime \prime }\cos^{2}\theta_{1}{}^{\prime }\cos\theta^{\prime }+3A_{0001}{{}^{\prime }}^{2}A_{1000}{}^{\prime }A_{3000}{}^{\prime \prime }\cos^{2}\theta_{1}{}^{\prime }\\ \;+A_{0002}{}^{\prime }A_{1001}{}^{\prime \prime }\cos^{2}\theta_{1}{}^{\prime }\cos\theta^{\prime }+2A_{1000}{}^{\prime }A_{0002}{}^{\prime }A_{2000}{}^{\prime \prime }\cos^{2}\theta_{1}{}^{\prime }+2A_{0001}{}^{\prime }A_{1001}{}^{\prime }A_{2000}{}^{\prime \prime }\cos^{2}\theta_{1}{}^{\prime }\\ \;-\left(\frac{1}{2R_{1}}-\frac{\cos\theta_{1}{}^{\prime }}{r}\right)\times \left(\frac{r_{2}A_{1000}{}^{\prime \prime }\cos\theta_{1}{}^{\prime }\cos^{2}\theta \sec\theta^{\prime }\sec\theta_{3}}{r^{2}}+A_{1001}{}^{\prime }A_{1000}{}^{\prime \prime }\sin\theta_{1}{}^{\prime }\right)\\ \;-\frac{r_{2}A_{1000}{}^{\prime \prime }\sec\theta^{\prime }\sec\theta_{2}}{r^{3}}\left(\cos^{2}\theta +\sin\theta_{1}{}^{\prime }\cos\theta_{1}{}^{\prime }\left(2\sin\theta \cos\theta +\Gamma_{2}\cos^{3}\theta \sec\theta^{\prime }\right)\right)\\ \;+\left(2A_{1000}{}^{\prime }A_{0001}{}^{\prime }A_{2000}{}^{\prime \prime }+A_{0001}{}^{\prime }A_{1001}{}^{\prime \prime }\cos\theta^{\prime }+\frac{A_{0001}{}^{\prime }A_{0001}{}^{\prime \prime }\sin\theta^{\prime }\cos\theta_{2}}{r^{\prime }}\right)\times (\frac{\sin\theta_{1}{}^{\prime }\cos\theta_{1}{}^{\prime }}{r}\\ \;-\frac{\sin\theta_{1}{}^{\prime }}{2R_{1}})+\frac{2A_{0001}{{}^{\prime }}^{2}A_{2000}{}^{\prime \prime }\sin\theta_{1}{}^{\prime }\cos\theta_{1}{}^{\prime }\cos\theta }{r}+\frac{\sin\theta^{\prime }\cos\theta_{2}}{r^{\prime }}(A_{0002}{}^{\prime }A_{0001}{}^{\prime \prime }\cos^{2}\theta_{1}{}^{\prime }\\ \;+A_{0001}{{}^{\prime }}^{2}A_{1001}{}^{\prime \prime })-\frac{A_{0001}{{}^{\prime }}^{2}A_{0001}{}^{\prime \prime }\cos^{2}\theta_{1}{}^{\prime }\sin\theta^{\prime }\sin\theta_{2}}{r^{\prime }}\times \left(\frac{1}{2R_{2}}-\frac{\cos\theta_{2}}{r^{\prime }}\right), \end{array}$$

(A16) $$\begin{array}{l} C_{0201}=A_{0201}{}^{\prime }A_{1000}{}^{\prime \prime }\cos\theta_{1}{}^{\prime }+A_{0001}{}^{\prime }A_{1020}{}^{\prime \prime }\cos\theta_{1}{}^{\prime }+2A_{0001}{}^{\prime }A_{0200}{}^{\prime }A_{2000}{}^{\prime \prime }\cos^{2}\theta_{1}{}^{\prime }+A_{0001}{}^{\prime }A_{1110}{}^{\prime \prime }\\ \;\times B_{0100}{}^{\prime }\cos\theta_{1}{}^{\prime }+A_{0110}{}^{\prime \prime }B_{0101}{}^{\prime }\cos\theta_{1}{}^{\prime }+A_{0001}{}^{\prime }A_{1200}{}^{\prime \prime }B_{0100}{{}^{\prime }}^{2}\cos\theta_{1}{}^{\prime }+2A_{0200}{}^{\prime \prime }B_{0100}{}^{\prime }B_{0101}{}^{\prime }\\ \;\times \cos\theta_{1}{}^{\prime }-\frac{A_{1000}{}^{\prime \prime }\sin\theta_{1}{}^{\prime }}{r_{2}}\left(A_{0110}{}^{\prime }+\frac{A_{0001}{}^{\prime }\sin\theta }{2R}\right)+\frac{r_{2}\sin\theta_{1}{}^{\prime }}{r^{2}}\left(A_{0110}{}^{\prime }+2A_{0200}{}^{\prime \prime }B_{0100}{}^{\prime }\right)\\ \;+\frac{A_{0001}{}^{\prime }\cos\theta_{1}{}^{\prime }}{2R}\left(\frac{A_{0001}{}^{\prime \prime }\cos\theta^{\prime }\cos\theta_{2}}{r^{\prime }}-A_{1001}{}^{\prime \prime }\sin\theta^{\prime }\right), \end{array}$$

(A17) $$\begin{array}{l} C_{0111}=A_{0111}{}^{\prime }A_{1000}{}^{\prime \prime }\cos\theta_{1}{}^{\prime }+2A_{0001}{}^{\prime }A_{0110}{}^{\prime }A_{2000}{}^{\prime \prime }\cos\theta_{1}{}^{\prime }+A_{0001}{}^{\prime }A_{1110}{}^{\prime \prime }B_{0010}{}^{\prime }\cos\theta_{1}{}^{\prime }+2A_{0001}{}^{\prime }\\ \;\times A_{1200}{}^{\prime \prime }B_{0010}{}^{\prime }B_{0100}{}^{\prime }\cos\theta_{1}{}^{\prime }+\frac{A_{1000}{}^{\prime \prime }\sin\theta_{1}{}^{\prime }}{r}\left(-2A_{0020}{}^{\prime }+A_{0110}{}^{\prime }\right)+\frac{2r_{2}A_{0200}{}^{\prime \prime }B_{0010}{}^{\prime }\sin\theta_{1}{}^{\prime }}{r^{2}}\\ \;+\left(A_{0110}{}^{\prime \prime }+2A_{0200}{}^{\prime \prime }B_{0100}{}^{\prime }\right)\left(-\frac{r_{2}\sin\theta_{1}{}^{\prime }}{r^{2}}+B_{0011}{}^{\prime }\cos\theta_{1}{}^{\prime }\right)+2A_{0200}{}^{\prime \prime }B_{0010}{}^{\prime }B_{0101}{}^{\prime }\cos\theta_{1}{}^{\prime }, \end{array}$$

(A18) $$\begin{array}{l} C_{0003}=A_{0003}{}^{\prime }A_{1000}{}^{\prime \prime }\cos^{3}\theta_{1}{}^{\prime }+A_{0001}{{}^{\prime }}^{3}A_{3000}{}^{\prime \prime }\cos^{3}\theta_{1}{}^{\prime }+2A_{0001}{}^{\prime }A_{0002}{}^{\prime }A_{2000}{}^{\prime \prime }\cos^{3}\theta_{1}{}^{\prime }\\ \;+\frac{A_{0001}{}^{\prime }A_{1000}{}^{\prime \prime }\cos^{2}\theta_{1}{}^{\prime }}{2R_{1}r}-\frac{A_{0001}{}^{\prime }A_{1000}{}^{\prime \prime }\sin^{2}\theta_{1}{}^{\prime }}{r}\times \left(\frac{1}{2R_{1}}-\frac{\cos\theta_{1}{}^{\prime }}{r}\right)\\ \;+\left(\frac{1}{2R_{1}}-\frac{\cos\theta_{1}{}^{\prime }}{r}\right)\times \left(2\sin\theta_{1}{}^{\prime }\cos\theta_{1}{}^{\prime }\left(A_{0002}{}^{\prime }A_{1000}{}^{\prime \prime }+A_{0001}{{}^{\prime }}^{2}A_{2000}{}^{\prime \prime }\right)\right), \end{array}$$

(A19) $$D_{0100}=B_{0100}{}^{\prime }B_{0100}{}^{\prime \prime }-\frac{r_{2}}{r^{\prime }},$$

(A20) $$D_{0010}=B_{0010}{}^{\prime }B_{0100}{}^{\prime \prime },$$

(A21) $$\begin{array}{l} D_{1100}=B_{1100}{}^{\prime }B_{0100}{}^{\prime \prime }+A_{1000}{}^{\prime }B_{1010}{}^{\prime \prime }+A_{1000}{}^{\prime }B_{0100}{}^{\prime }B_{1100}{}^{\prime \prime }+B_{0011}{}^{\prime \prime }\cos\theta^{\prime }\\ \;+B_{0100}{}^{\prime }B_{0101}{}^{\prime \prime }\cos\theta^{\prime }-\frac{r_{2}\sin\theta^{\prime }}{r^{\prime }{}^{2}}\left(1-B_{0100}{}^{\prime }\right), \end{array}$$

(A22) $$D_{1010}=B_{1010}{}^{\prime }B_{0100}{}^{\prime \prime }+A_{1000}{}^{\prime }B_{0010}{}^{\prime }B_{1100}{}^{\prime \prime }+B_{0010}{}^{\prime }B_{0101}{}^{\prime \prime }\cos\theta^{\prime }+\frac{r_{2}B_{0010}{}^{\prime }\sin\theta^{\prime }}{r^{\prime }{}^{2}},$$

(A23) $$D_{0101}=B_{0101}{}^{\prime }B_{0100}{}^{\prime \prime }\cos\theta_{1}{}^{\prime }+A_{0001}{}^{\prime }B_{1010}{}^{\prime \prime }\cos\theta_{1}{}^{\prime }+A_{0001}{}^{\prime }B_{0100}{}^{\prime }B_{1100}{}^{\prime \prime }\cos\theta_{1}{}^{\prime }+\frac{r^{\prime }B_{0100}{}^{\prime \prime }\sin\theta_{1}{}^{\prime }}{r^{2}},$$

(A24) $$D_{0011}=B_{0011}{}^{\prime }B_{0100}{}^{\prime \prime }\cos\theta_{1}{}^{\prime }+A_{0001}{}^{\prime }B_{0010}{}^{\prime }B_{1100}{}^{\prime \prime }\cos\theta_{1}{}^{\prime }-\frac{r^{\prime }B_{0100}{}^{\prime \prime }\sin\theta_{1}{}^{\prime }}{r^{2}},$$

(A25) $$\begin{array}{l} D_{2100}=B_{2100}{}^{\prime }B_{0100}{}^{\prime \prime }+A_{2000}{}^{\prime }B_{1010}{}^{\prime \prime }+A_{2000}{}^{\prime }B_{0100}{}^{\prime }B_{1100}{}^{\prime \prime }+A_{1000}{}^{\prime }B_{1100}{}^{\prime }B_{1100}{}^{\prime \prime }+B_{0100}{}^{\prime }B_{0102}{}^{\prime \prime }\cos^{2}\theta^{\prime }\\ \;+A_{1000}{{}^{\prime }}^{2}B_{0100}{}^{\prime }B_{2100}{}^{\prime \prime }+B_{1100}{}^{\prime }B_{0101}{}^{\prime \prime }\cos\theta^{\prime }+B_{0012}{}^{\prime \prime }\cos^{2}\theta^{\prime }\;+A_{1000}{}^{\prime }B_{1011}{}^{\prime \prime }\cos\theta^{\prime }\\ \;+A_{1000}{{}^{\prime }}^{2}B_{2010}{}^{\prime \prime }+A_{1000}{}^{\prime }B_{0100}{}^{\prime }B_{1101}{}^{\prime \prime }\cos\theta^{\prime }+\frac{r_{2}B_{1100}{}^{\prime }\sin\theta^{\prime }}{r^{\prime }{}^{2}}-B_{0100}{}^{\prime }B_{0101}{}^{\prime \prime }\sin\theta^{\prime }\\ \;\times \left(\frac{1}{2R}-\frac{\cos\theta^{\prime }}{r^{\prime }}\right)-\frac{r_{2}\left(1-B_{1100}{}^{\prime }\right)}{r^{\prime }{}^{2}}\times (\frac{\sin^{2}\theta^{\prime }}{r^{\prime }}+\frac{A_{1000}{}^{\prime }\sin\theta^{\prime }\sin\theta_{2}}{r^{\prime }}+\frac{\cos\theta^{\prime }}{2R})\\ \;+\frac{A_{1000}{}^{\prime }\sin\theta^{\prime }}{r^{\prime }}(B_{1010}{}^{\prime \prime }-B_{0100}{}^{\prime }B_{1010}{}^{\prime \prime }\;+B_{0100}{}^{\prime }B_{0101}{}^{\prime \prime }\cos\theta_{2}), \end{array}$$

(A26) $$\begin{array}{l} D_{2010}=B_{2010}{}^{\prime }B_{0100}{}^{\prime \prime }+A_{2000}{}^{\prime }B_{0010}{}^{\prime }B_{1100}{}^{\prime \prime }+A_{1000}{}^{\prime }B_{1010}{}^{\prime }B_{1100}{}^{\prime \prime }+A_{1000}{{}^{\prime }}^{2}B_{0010}{}^{\prime }B_{2100}{}^{\prime \prime }\\ \;+B_{1010}{}^{\prime }B_{0101}{}^{\prime \prime }\cos\theta^{\prime }+A_{1000}{}^{\prime }B_{0010}{}^{\prime }B_{1101}{}^{\prime \prime }\cos\theta^{\prime }+B_{0010}{}^{\prime }B_{0102}{}^{\prime \prime }\cos^{2}\theta^{\prime }\\ \;-\frac{B_{0010}{}^{\prime }B_{0101}{}^{\prime \prime }\sin\theta^{\prime }}{2R}+\frac{r_{2}B_{0010}{}^{\prime }\sin\theta^{\prime }}{r^{\prime }{}^{3}}\left(\sin\theta^{\prime }+A_{1000}{}^{\prime }\sin\theta_{2}\right)\\ \;+\frac{r_{2}B_{0010}{}^{\prime }\cos\theta^{\prime }}{2Rr^{\prime }{}^{2}}+\frac{r_{2}B_{1010}{}^{\prime }\sin\theta^{\prime }}{r^{\prime }{}^{2}}-\frac{B_{0010}{}^{\prime }\sin\theta^{\prime }}{r^{\prime }}(A_{1000}{}^{\prime }B_{1010}{}^{\prime }-B_{0101}{}^{\prime \prime }\\ \;\times \cos\theta^{\prime }-A_{1000}{}^{\prime }B_{0101}{}^{\prime \prime }\cos\theta_{2}), \end{array}$$

(A27) $$\begin{array}{l} D_{1101}=B_{1101}{}^{\prime }B_{0100}{}^{\prime \prime }\cos\theta_{1}{}^{\prime }+A_{1000}{}^{\prime }B_{0101}{}^{\prime }B_{1100}{}^{\prime \prime }\cos\theta_{1}{}^{\prime }+A_{0001}{}^{\prime }B_{0100}{}^{\prime }B_{1101}{}^{\prime \prime }\cos\theta_{1}{}^{\prime }\cos\theta^{\prime }\\ \;+A_{1001}{}^{\prime }B_{0100}{}^{\prime }B_{1100}{}^{\prime \prime }\cos\theta_{1}{}^{\prime }+A_{0001}{}^{\prime }B_{1100}{}^{\prime }B_{1100}{}^{\prime \prime }\cos\theta_{1}{}^{\prime }+2A_{0001}{}^{\prime }A_{1000}{}^{\prime }B_{2010}{}^{\prime \prime }\cos\theta_{1}{}^{\prime }\\ \;+2A_{0001}{}^{\prime }A_{1000}{}^{\prime }B_{0100}{}^{\prime }B_{2100}{}^{\prime \prime }\cos\theta_{1}{}^{\prime }+A_{0001}{}^{\prime }B_{1011}{}^{\prime \prime }\cos\theta_{1}{}^{\prime }\cos\theta^{\prime }+A_{1001}{}^{\prime }B_{1010}{}^{\prime \prime }\cos\theta_{1}{}^{\prime }\\ \;+B_{0101}{}^{\prime }B_{0101}{}^{\prime \prime }\cos\theta_{1}{}^{\prime }\cos\theta^{\prime }+\frac{B_{0100}{}^{\prime \prime }\sin\theta_{1}{}^{\prime }}{r}\left(-B_{1010}{}^{\prime }+B_{0101}{}^{\prime }\cos\theta +\frac{r^{\prime }\sin\theta }{r^{2}}\right)\\ \;+\frac{A_{0001}{}^{\prime }\sin\theta_{1}{}^{\prime }\cos\theta }{r}\left(B_{1010}{}^{\prime \prime }+B_{0100}{}^{\prime }B_{1100}{}^{\prime \prime }\right)+\left(A_{1000}{}^{\prime }B_{1100}{}^{\prime \prime }+B_{0101}{}^{\prime \prime }\cos\theta^{\prime }\right)\\ \;\times \frac{r^{\prime }\sin\theta_{1}{}^{\prime }}{r^{2}}+\frac{A_{0001}{}^{\prime }\cos\theta_{1}{}^{\prime }\sin\theta^{\prime }}{r^{\prime }}\left(B_{1010}{}^{\prime }-B_{0100}{}^{\prime }B_{1010}{}^{\prime \prime }+B_{0100}{}^{\prime }B_{0101}{}^{\prime \prime }\cos\theta_{2}\right)\\ \;+\frac{\sin\theta^{\prime }}{r^{\prime }{}^{2}}\left(\frac{r^{\prime }r_{2}\sin\theta_{1}{}^{\prime }}{r^{2}}+r_{2}B_{0101}{}^{\prime }\cos\theta_{1}{}^{\prime }-\frac{r_{2}A_{0001}{}^{\prime }\cos\theta_{1}{}^{\prime }\sin\theta_{2}}{r^{\prime }}\left(1-B_{0100}{}^{\prime }\right)\right), \end{array}$$

(A28) $$\begin{array}{l} \;\;D_{1011}=A_{0001}{}^{\prime }B_{1010}{}^{\prime }B_{1100}{}^{\prime \prime }\cos\theta_{1}{}^{\prime }+A_{1001}{}^{\prime }B_{0010}{}^{\prime }B_{1100}{}^{\prime \prime }\cos\theta_{1}{}^{\prime }+A_{1000}{}^{\prime }B_{0011}{}^{\prime }B_{1100}{}^{\prime \prime }\cos\theta_{1}{}^{\prime }\\ \;+B_{1011}{}^{\prime }B_{0100}{}^{\prime \prime }\cos\theta_{1}{}^{\prime }+B_{0011}{}^{\prime }B_{0101}{}^{\prime \prime }\cos\theta_{1}{}^{\prime }\cos\theta^{\prime }+A_{0001}{}^{\prime }B_{0010}{}^{\prime }B_{1101}{}^{\prime \prime }\cos\theta_{1}{}^{\prime }\cos\theta^{\prime }\\ \;+2A_{0001}{}^{\prime }A_{1000}{}^{\prime }B_{0010}{}^{\prime }B_{2100}{}^{\prime \prime }\cos\theta_{1}{}^{\prime }+\frac{\sin\theta_{1}{}^{\prime }}{r}\left(B_{1010}{}^{\prime }B_{0100}{}^{\prime \prime }+A_{0001}{}^{\prime }B_{0010}{}^{\prime }B_{1100}{}^{\prime \prime }\cos\theta \right)\\ \;-\frac{r^{\prime }\sin\theta_{1}{}^{\prime }}{r^{2}}\left(A_{1000}{}^{\prime }B_{1100}{}^{\prime \prime }+B_{0101}{}^{\prime \prime }\cos\theta^{\prime }\right)-\frac{r^{\prime }}{r^{3}}\left(\sin\theta +\cos^{2}\theta \sec\theta^{\prime }\tan\theta_{2}\right)\\ \;\times B_{0100}{}^{\prime \prime }\sin\theta_{1}{}^{\prime }-\frac{A_{0001}{}^{\prime }B_{0010}{}^{\prime }\cos\theta_{1}{}^{\prime }\sin\theta^{\prime }}{r^{\prime }}\left(B_{1010}{}^{\prime \prime }-B_{0101}{}^{\prime \prime }\cos\theta_{2}\right)+\frac{r_{2}\sin\theta^{\prime }}{r^{\prime }{}^{2}}\\ \;\times \left(-\frac{r^{\prime }\sin\theta_{1}{}^{\prime }}{r^{2}}+B_{0011}{}^{\prime }\cos\theta_{1}{}^{\prime }+\frac{A_{0001}{}^{\prime }B_{0010}{}^{\prime }\cos\theta_{1}{}^{\prime }\sin\theta_{2}}{r^{\prime }}\right), \end{array}$$

(A29) $$\begin{array}{l} D_{0300}=B_{0300}{}^{\prime }B_{0100}{}^{\prime \prime }+B_{0100}{}^{\prime }B_{0120}{}^{\prime \prime }+B_{0100}{{}^{\prime }}^{2}B_{0210}{}^{\prime \prime }+B_{0100}{{}^{\prime }}^{3}B_{0300}{}^{\prime \prime }+A_{0200}{}^{\prime }B_{0100}{}^{\prime }B_{1100}{}^{\prime \prime }\\ \;+A_{0200}{}^{\prime }B_{1010}{}^{\prime \prime }-\frac{1}{2R}\left(B_{0100}{}^{\prime }B_{0120}{}^{\prime \prime }\sin\theta^{\prime }+\frac{r_{2}\cos\theta^{\prime }}{r^{\prime }{}^{2}}\left(1-B_{0100}{}^{\prime }\right)\right), \end{array}$$

(A30) $$\begin{array}{l} D_{0300}=B_{0300}{}^{\prime }B_{0100}{}^{\prime \prime }+B_{0100}{}^{\prime }B_{0120}{}^{\prime \prime }+B_{0100}{{}^{\prime }}^{2}B_{0210}{}^{\prime \prime }+B_{0100}{{}^{\prime }}^{3}B_{0300}{}^{\prime \prime }+A_{0200}{}^{\prime }B_{0100}{}^{\prime }B_{1100}{}^{\prime \prime }\\ \;+A_{0200}{}^{\prime }B_{1010}{}^{\prime \prime }-\frac{1}{2R}\left(B_{0100}{}^{\prime }B_{0120}{}^{\prime \prime }\sin\theta^{\prime }+\frac{r_{2}\cos\theta^{\prime }}{r^{\prime }{}^{2}}\left(1-B_{0100}{}^{\prime }\right)\right), \end{array}$$

(A31) $$\begin{array}{l} D_{0120}=B_{0120}{}^{\prime }B_{0100}{}^{\prime \prime }+A_{0110}{}^{\prime }B_{0010}{}^{\prime }B_{1100}{}^{\prime \prime }+A_{0020}{}^{\prime }B_{0100}{}^{\prime }B_{1100}{}^{\prime \prime }+B_{0010}{{}^{\prime }}^{2}B_{0210}{}^{\prime \prime }+A_{0020}{}^{\prime }B_{1010}{}^{\prime \prime }\\ \;+3B_{0100}{}^{\prime }B_{0010}{{}^{\prime }}^{2}B_{0300}{}^{\prime \prime }-\frac{\sin\theta_{1}{}^{\prime }}{2R_{1}}\left(B_{0101}{}^{\prime }B_{0100}{}^{\prime \prime }+A_{0001}{}^{\prime }\left(B_{1010}{}^{\prime \prime }+B_{0100}{}^{\prime }B_{1100}{}^{\prime \prime }\right)\right)\\ \;-\frac{r^{\prime }B_{0100}{}^{\prime \prime }\cos\theta_{1}{}^{\prime }}{2R_{1}r^{2}}, \end{array}$$

(A32) $$\begin{array}{l} D_{0102}=A_{0001}{{}^{\prime }}^{2}B_{2010}{}^{\prime \prime }\cos^{2}\theta_{1}{}^{\prime }+A_{0001}{{}^{\prime }}^{2}B_{0100}{}^{\prime }B_{2100}{}^{\prime \prime }\cos^{2}\theta_{1}{}^{\prime }+A_{0002}{}^{\prime }B_{1010}{}^{\prime \prime }\cos^{2}\theta_{1}{}^{\prime }\\ \;+A_{0001}{}^{\prime }B_{0101}{}^{\prime }B_{1100}{}^{\prime \prime }\cos^{2}\theta_{1}{}^{\prime }-\frac{1}{2R_{1}}\left(B_{0100}{}^{\prime }B_{2100}{}^{\prime \prime }\sin\theta_{1}{}^{\prime }-\frac{r^{\prime }B_{0100}{}^{\prime \prime }\cos\theta_{1}{}^{\prime }}{r^{2}}\right)\\ \;+\;B_{0102}{}^{\prime }B_{0100}{}^{\prime \prime }+A_{0002}{}^{\prime }B_{0100}{}^{\prime }B_{1100}{}^{\prime \prime }\cos^{2}\theta_{1}{}^{\prime }+\frac{r^{\prime }A_{0001}{}^{\prime }B_{1100}{}^{\prime \prime }\sin\theta_{1}{}^{\prime }\cos\theta_{1}{}^{\prime }}{r^{2}}\\ \;+\left(\frac{\sin\theta_{1}{}^{\prime }\cos\theta_{1}{}^{\prime }}{r}-\frac{\sin\theta_{1}{}^{\prime }}{2R_{1}}\right)\left(A_{0001}{}^{\prime }B_{1010}{}^{\prime \prime }+A_{0001}{}^{\prime }B_{0100}{}^{\prime }B_{1100}{}^{\prime \prime }\right), \end{array}$$

(A33) $$D_{0030}=B_{0100}{}^{\prime \prime }\left(B_{0030}{}^{\prime }-\frac{r^{\prime }\cos\theta_{1}{}^{\prime }}{2R_{1}r^{2}}\right)+B_{0010}{}^{\prime }\left(B_{0010}{{}^{\prime }}^{2}B_{0300}{}^{\prime \prime }+A_{0020}{}^{\prime }B_{1100}{}^{\prime \prime }\right)-\frac{A_{0001}{}^{\prime }B_{0010}{}^{\prime }B_{1100}{}^{\prime \prime }\sin\theta_{1}{}^{\prime }}{2R_{1}},$$

(A34) $$\begin{array}{l} D_{0012}=B_{0012}{}^{\prime }B_{0100}{}^{\prime \prime }\cos^{2}\theta_{1}{}^{\prime }+A_{0002}{}^{\prime }B_{0010}{}^{\prime }B_{1100}{}^{\prime \prime }\cos^{2}\theta_{1}{}^{\prime }+A_{0001}{{}^{\prime }}^{2}B_{0010}{}^{\prime }B_{2100}{}^{\prime \prime }\cos^{2}\theta_{1}{}^{\prime }+A_{0001}{}^{\prime }B_{0011}{}^{\prime }\\ \;\times B_{1100}{}^{\prime \prime }\cos^{2}\theta_{1}{}^{\prime }+\frac{r^{\prime }}{r^{2}}(B_{0100}{}^{\prime \prime }(\left(\frac{1}{2R_{1}}-\frac{2\cos\theta_{1}{}^{\prime }}{r}\right)\sin\theta_{1}{}^{\prime }\cos\theta \sec\theta^{\prime }\tan\theta_{2}-\frac{\cos\theta_{1}{}^{\prime }}{2R_{1}}\\ \;\;-\frac{\sin^{2}\theta_{1}{}^{\prime }}{r})-A_{0002}{}^{\prime }B_{1100}{}^{\prime \prime }\sin\theta_{1}{}^{\prime }\cos\theta_{1}{}^{\prime })+A_{0001}{}^{\prime }B_{0010}{}^{\prime }B_{1100}{}^{\prime \prime }(\frac{\sin\theta_{1}{}^{\prime }\cos\theta_{1}{}^{\prime }}{r}-\frac{\sin\theta_{1}{}^{\prime }}{2R_{1}}). \end{array}$$

## Appendix B. Aberration Coefficients *E_hijk_* and *F_hijk_*

(A35) $$E_{1000}=A_{1000}C_{1000}+C_{0001},$$

(A36) $$E_{0001}=A_{0001}C_{1000},$$

(A37) $$E_{2000}=A_{2000}C_{1000}+A_{1000}{}^{2}C_{2000}+A_{1000}C_{1001}+C_{0002},$$

(A38) $$E_{1001}=A_{1001}C_{1000}+2A_{0001}A_{1000}C_{2000}+A_{0001}C_{1001},$$

(A39) $$E_{0200}=A_{0200}C_{1000}+B_{0100}{}^{2}C_{0200}+B_{0100}C_{0110}+C_{0020},$$

(A40) $$E_{0110}=A_{0110}C_{1000}+2B_{0100}B_{0010}C_{0200}+B_{0010}C_{0110},$$

(A41) $$E_{0020}=A_{0020}C_{1000}+B_{0010}{}^{2}C_{0200},$$

(A42) $$E_{0002}=A_{0002}C_{1000}+A_{0001}{}^{2}C_{2000},$$

(A43) $$E_{3000}=A_{3000}C_{1000}+2A_{1000}A_{2000}C_{2000}+A_{2000}C_{1001}+A_{1000}{}^{3}C_{3000}+A_{1000}{}^{2}C_{2001}+A_{1000}C_{1002}+C_{0003},$$

(A44) $$\begin{array}{l} E_{2001}=A_{2001}C_{1000}+2A_{1000}A_{1001}C_{2000}+2A_{0001}A_{2000}C_{2000}+A_{1001}C_{1001}\\ \;+3A_{0001}A_{1000}{}^{2}C_{3000}+2A_{1000}A_{0001}C_{2001}+A_{0001}C_{1002}, \end{array}$$

(A45) $$\begin{array}{l} E_{1200}=A_{1200}C_{1000}+2A_{1000}A_{0200}C_{2000}+A_{0200}C_{1001}+2B_{1100}B_{0100}C_{0200}+B_{1100}C_{0110}+A_{1000}C_{1020}\\ \;+A_{1000}B_{0100}{}^{2}C_{1200}+A_{1000}B_{0100}C_{1110}+B_{0100}{}^{2}C_{0201}+B_{0100}C_{0111}+C_{0021}, \end{array}$$

(A46) $$\begin{array}{l} E_{1110}=A_{1110}C_{1000}+2A_{1000}A_{0110}C_{2000}+A_{0110}C_{1001}+2B_{1010}B_{0100}C_{0200}+2B_{1100}B_{0010}C_{0200}+B_{1010}C_{0110}\\ \;+2A_{1000}B_{0010}B_{0100}C_{1200}+A_{1000}B_{0010}C_{1110}+2B_{0010}B_{0100}C_{0201}+B_{0010}C_{0111}, \end{array}$$

(A47) $$E_{1020}=A_{1020}C_{1000}+2A_{1000}A_{0020}C_{2000}+A_{0020}C_{1001}+2B_{0010}B_{1010}C_{0200}+A_{1000}B_{0010}{}^{2}C_{1200}+B_{0010}{}^{2}C_{0201},$$

(A48) $$\begin{array}{l} E_{1002}=A_{1002}C_{1000}+2A_{1000}A_{0002}C_{2000}+2A_{0001}A_{1001}C_{2000}+A_{0002}C_{1001}+3A_{1000}A_{0001}{}^{2}C_{3000}\\ \;+A_{0001}{}^{2}C_{2001}, \end{array}$$

(A49) $$\begin{array}{l} E_{0201}=A_{0201}C_{1000}+2A_{0001}A_{0200}C_{2000}+2B_{0101}B_{0100}C_{0200}+B_{0101}C_{0110}\\ \;+A_{0001}B_{0100}{}^{2}C_{1200}+A_{0001}B_{0100}C_{1110}+A_{0001}C_{1020}, \end{array}$$

(A50) $$\begin{array}{l} E_{0111}=A_{0111}C_{1000}+2A_{0110}A_{0001}C_{2000}+2B_{0100}B_{0011}C_{0200}+2B_{0010}B_{0101}C_{0200}\\ \;+B_{0011}C_{0110}+2A_{0001}B_{0100}B_{0010}C_{1200}+A_{0001}B_{0010}C_{1110}, \end{array}$$

(A51) $$E_{0021}=A_{0021}C_{1000}+2A_{0020}A_{0001}C_{2000}+2B_{0010}B_{0011}C_{0200}+A_{0001}B_{0010}{}^{2}C_{1200},$$

(A52) $$E_{0003}=A_{0003}C_{1000}+2A_{0002}A_{0001}C_{2000}+A_{0001}{}^{3}C_{3000},$$

(A53) $$F_{0100}=D_{0010}+B_{0100}D_{0100},$$

(A54) $$F_{0010}=B_{0010}D_{0100},$$

(A55) $$F_{1100}=B_{1100}D_{0100}+A_{1000}B_{0100}D_{1100}+A_{1000}D_{1010}+B_{0100}D_{0101}+D_{0011},$$

(A56) $$F_{1010}=B_{1010}D_{0100}+A_{1000}B_{0010}D_{1100}+B_{0010}D_{0101},$$

(A57) $$F_{0101}=B_{0101}D_{0100}+A_{0001}B_{0100}D_{1100}+A_{0001}D_{1010},$$

(A58) $$F_{0011}=B_{0011}D_{0100}+A_{0001}B_{0010}D_{1100},$$

(A59) $$\begin{array}{l} F_{2100}=B_{2100}D_{0100}+A_{1000}B_{1100}D_{1100}+A_{2000}B_{0100}D_{1100}+A_{2000}D_{1010}+A_{1000}{}^{2}B_{0100}D_{2100}\\ \;+B_{1100}D_{0101}+A_{1000}{}^{2}D_{2010}+A_{1000}B_{0100}D_{1101}+A_{1000}D_{1011}+B_{0100}D_{0102}+D_{0012}, \end{array}$$

(A60) $$\begin{array}{l} F_{2010}=B_{2010}D_{0100}+A_{1000}B_{1010}D_{1100}+A_{2000}B_{0010}D_{1100}+B_{1010}D_{0101}+A_{1000}{}^{2}B_{0010}D_{2100}\\ \;+A_{1000}B_{0010}D_{1101}+B_{0010}D_{0102}, \end{array}$$

(A61) $$\begin{array}{l} F_{1101}=B_{1101}D_{0100}+A_{1000}B_{0101}D_{1100}+A_{0001}B_{1100}D_{1100}+A_{1001}B_{0100}D_{1100}+A_{0001}B_{0100}D_{1101}\\ \;+B_{0101}D_{0101}+A_{1001}D_{1010}+2A_{1000}A_{0001}B_{0100}D_{2100}+2A_{1000}A_{0001}D_{2010}+A_{0001}D_{1011}, \end{array}$$

(A62) $$\begin{array}{l} F_{1011}=B_{1011}D_{0100}+A_{1000}B_{0011}D_{1100}+A_{0001}B_{1010}D_{1100}+A_{1001}B_{0010}D_{1100}\\ \;+B_{0011}D_{0101}+2A_{1000}A_{0001}B_{0010}D_{2100}+A_{0001}B_{0010}D_{1101}, \end{array}$$

(A63) $$\begin{array}{l} F_{0210}=B_{0210}D_{0100}+A_{0110}B_{0100}D_{1100}+A_{0200}B_{0010}D_{1100}+A_{0110}D_{1010}+3B_{0010}B_{0100}{}^{2}D_{0300}\\ \;+2B_{0100}B_{0010}D_{0210}+B_{0010}D_{0120}, \end{array}$$

(A64) $$F_{0300}=B_{0300}D_{0100}+A_{0200}B_{0100}D_{1100}+A_{0200}D_{1010}+B_{0100}{}^{3}D_{0300}+B_{0100}{}^{2}D_{0210}+B_{0100}D_{0120}+D_{0030},$$

(A65) $$F_{0120}=B_{0120}D_{0100}+A_{0110}B_{0010}D_{1100}+A_{0020}B_{0100}D_{1100}+A_{0020}D_{1010}+3B_{0100}B_{0010}{}^{2}D_{0300}+B_{0010}{}^{2}D_{0210},$$

(A66) $$F_{0102}=B_{0102}D_{0100}+A_{0001}B_{0101}D_{1100}+A_{0002}B_{0100}D_{1100}+A_{0002}D_{1010}+A_{0001}{}^{2}B_{0100}D_{2100}+A_{0001}{}^{2}D_{2010},$$

(A67) $$F_{0030}=B_{0030}D_{0100}+A_{0020}B_{0010}D_{1100}+B_{0010}{}^{3}D_{0300},$$

(A68) $$F_{0012}=B_{0012}D_{0100}+A_{0002}B_{0010}D_{1100}+A_{0001}B_{0011}D_{1100}+A_{0001}{}^{2}B_{0010}D_{2100},$$
